# Geographic extent of introgression in *Sebastes mentella* and its effect on genetic population structure

**DOI:** 10.1111/eva.12429

**Published:** 2016-10-22

**Authors:** Atal Saha, Torild Johansen, Rasmus Hedeholm, Einar E. Nielsen, Jon‐Ivar Westgaard, Lorenz Hauser, Benjamin Planque, Steven X. Cadrin, Jesper Boje

**Affiliations:** ^1^ Tromsø DepartmentInstitute of Marine ResearchTromsøNorway; ^2^ Greenland Institute of Natural ResourcesNuukGreenland; ^3^ DTU Aqua – National Institute of Aquatic ResourcesCharlottenlundDenmark; ^4^ School of Aquatic and Fishery SciencesUniversity of WashingtonSeattleWAUSA; ^5^ Hjort Centre for Marine Ecosystem DynamicsBergenNorway; ^6^ School for Marine Science and TechnologyUniversity of Massachusetts DarmouthFairhavenMAUSA

**Keywords:** gene flow, hybrid zone, incipient speciation, oceanic, redfish

## Abstract

Genetic population structure is often used to identify management units in exploited species, but the extent of genetic differentiation may be inflated by geographic variation in the level of hybridization between species. We identify the genetic population structure of *Sebastes mentella* and investigate possible introgression within the genus by analyzing 13 microsatellites in 2,562 redfish specimens sampled throughout the North Atlantic. The data support an historical divergence between the “shallow” and “deep” groups, beyond the Irminger Sea where they were described previously. A third group*,* “slope,” has an extended distribution on the East Greenland Shelf, in addition to earlier findings on the Icelandic slope. Furthermore, *S. mentella* from the Northeast Arctic and Northwest Atlantic waters are genetically different populations. In both areas, interspecific introgression may influence allele frequency differences among populations. Evidence of introgression was found for almost all the identified *Sebastes* gene pools, but to a much lower extent than suggested earlier. Greenland waters appear to be a sympatric zone for many of the genetically independent *Sebastes* groups. This study illustrates that the identified groups maintain their genetic integrity in this region despite introgression.

## 1 Introduction

Identification of genetic heterogeneity and its application to define fishery management units are important for the sustainable utilization of living marine resources (Shaklee & Bentzen, 1998). Significant population genetic structure caused by a diverse array of factors has been described for many marine species (Gagnaire et al., 2015; Hauser & Carvalho, 2008; Salmenkova, 2011) despite the apparent lack of physical barriers to migration in the marine environment. Nevertheless, marine species usually display low genetic differentiation, indicating that some gene flow exists between apparently isolated groups. On the other hand, distinct species or populations may mate in a particular marine habitat while maintaining reproductive barrier in surrounding regions and thereby form so‐called hybrid zone (e.g., Nielsen, Hansen, Ruzzante, Meldrup, & Gronkjaer, 2003; Roques, Sevigny, & Bernatchez, 2001). Introgressive hybridization (i.e., introgression), where hybrids back‐cross with one of their parental genotypes (Baskett & Gomulkiewicz, 2011), may also influence allele frequency differences between populations causing intraspecific diversification (Artamonova et al., 2013; Roques et al., 2001). The effects of introgression on the genetic population structure of closely related marine fish species remains largely unexplored.

In the North Atlantic, redfishes (genus: *Sebastes*) are represented by four species: *Sebastes mentella* Travin 1951 (beaked redfish), *Sebastes norvegicus* Ascanius 1772 (golden redfish, previously called *S. marinus*), *Sebastes fasciatus* Storer 1854 (acadian redfish) and *Sebastes viviparus* Krøyer 1845 (Norwegian redfish). *Sebastes mentella* is the most economically important species of the genus. It displays a high degree of genetic population diversity across its distributional range. In the Irminger Sea, two distinct groups, “shallow (=shallow pelagic)” (50–550 m) and “deep (=deep pelagic)” (550–800 m), have been reported (Cadrin et al., 2010, 2011; Pampoulie & Daníelsdóttir, 2008; Stefansson et al., 2009). These two groups have been discriminated on the basis of morphological characteristics; the “deep” group has brighter red color, stouter appearance and larger size at sexual maturity (Magnusson & Magnusson, 1995). They have also been reported to show different rates of parasite infestation (Magnusson & Magnusson, 1995). Microsatellite DNA and morphological analyses suggested that these are two incipient species maintained by an ecological isolation mechanism, although evidence for hybridization was found (Stefánsson et al., 2009). The historical divergence between the “shallow” and “deep” groups was further supported by DNA analysis of the mitochondrial control region, microsatellites and the gene coding for the visual‐pigment rhodopsin (Shum, Pampoulie, Kristinsson, & Mariani, 2015; Shum, Pampoulie, Sacchi, & Mariani, 2014). Around Iceland, an additional “slope” component of *S. mentella* has been described (e.g., Cadrin et al., 2010). However, the genetic connectivity among these groups and their geographical distribution in the North Atlantic is not well understood.

*Sebastes* species coexist in different marine areas and overlap in their depth range, normally between 100 and 400 m (Barsukov, Litvinenko, & Serebryakov, 1984). For instance, *S. mentella* co‐occurs with *S. norvegicus* and *S. fasciatus* in the Northwest Atlantic (Roques et al., 2001), *S. norvegicus* in Greenland waters (ICES 2001), and *S. norvegicus* and *S. viviparus* in Icelandic and Norwegian waters (ICES 2001). Studies across the North Atlantic have indicated hybridization of *S. mentella* with *S. norvegicus* (Pampoulie & Daníelsdóttir, 2008), *S. viviparus* (Artamonova et al., 2013) and *S. fasciatus* (Roques et al., 2001). However, except for the evidence of hybridization in the Northwest Atlantic (Roques et al., 2001), the geographic extent of hybridization has not been well studied, which partly relates to the uncertainty about *Sebastes* mating grounds. The waters off East Greenland are assumed to be the main nursery area for *S. mentella* and *S. norvegicus* juveniles, which are believed to be extruded along the Reykjanes Ridge (Anderson, 1984; Magnusson & Johannesson, 1995). Morphological species identification remains uncertain (particularly for fish <25 cm) in these geographical regions, possibly because of unrecognized hybridization (Johansen, 2003). Hybridization has also been suspected to affect estimates of genetic population structure within species (Artamonova et al., 2013). Specifically, introgression with *S. viviparus* has been indicated as one of the main reasons for the apparent differentiation between the “deep” and “shallow” groups of *S. mentella* (Artamonova et al., 2013). The role of hybridization on the genetic population structure is therefore still uncertain.

Despite the importance of Greenland waters as habitat and fisheries area for *Sebastes* species, the genetic population structure of *S. mentella* in this region has not yet been described in sufficient detail for fisheries management. For instance, the “slope” group in the East Greenland and Iceland waters are assessed separately by the International Council for the Exploration of the Sea (ICES 2015a) although it is known that the stock identity is uncertain. ICES perceives this as an interim status until the stock structure of *S. mentella* on the East Greenland slope is better understood. The objectives of this study were: I) to examine the genetic structure of *S. mentella* in Greenland, Iceland, and Irminger Sea waters, II) to investigate the extent of introgression among co‐occurring *Sebastes* species/gene pools, and III) to assess whether introgression may influence apparent species and population structure in the region.

## 2 Materials and Methods

### 2.1 Sampling

**Figure 1:**
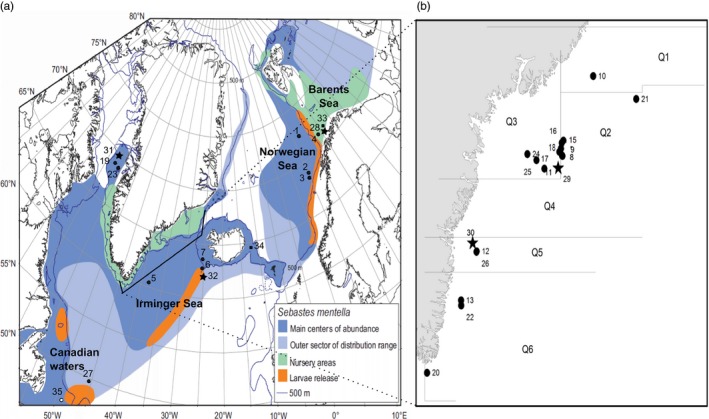
(a) Sampling locations of *Sebastes* spp. across the North Atlantic. For *Sebastes mentella*, the species distribution range, and larval release and nursery areas are presented (source: Planque et al. 2013). *S. mentella*,* Sebastes norvegicus*,* Sebastes viviparus*, and *Sebastes fasciatus* samples are indicated by numbers 1–27(●), 28–32 (★), 33–34 (■), and 35(○), respectively. (b) Samples from East Greenland waters are illustrated. Samples 5, 6, and 7 represent reference samples of *S. mentella* included from the EU REDFISH project (see Table 1)

In total, 35 samples (collections of fish) consisting of 2,562 redfish specimens were included in the study (Table 1). Nineteen *S. mentella* samples were collected during 2011 and 2012 from Greenland waters by commercial fishing vessels and research surveys. All the fish were caught by trawl. Samples were from different seasons (spring and fall) and life stages (juveniles and adults). Norwegian samples were collected from the shelf and pelagic waters in different seasons of the years (Table 1). *Sebastes mentella* reference samples were included from Canadian, Icelandic (representing the Icelandic shelf component) waters, and Irminger Sea from the EU REDFISH project (1998–2001). The Irminger Sea reference samples were characterized as “deep” and “shallow” *S. mentella* based on both morphological characters and sampling depths as described by Magnusson and Magnusson (1995). We included reference samples of *S. norvegicus*,* S. viviparus*, and *S. fasciatus* from Greenland, Iceland, Norwegian, and Canadian waters (Table 1, Figure 1) to study introgression with *S. mentella*. Species identification was conducted on board the ships based on morphological characters (e.g., body size, beak size, eye diameter, direction of spines in the pre‐operculum) as suggested by Barsukov et al. (1984). The adults and juveniles were categorized by length (Barsukov et al., 1984) [for instance, in *S. mentella*, adults ≥29 cm and juveniles = (4–28) cm].

**Table 1:** Details of the different *Sebastes* spp. samples analyzed. The sex ratio is given as % female and life stages as % adult (see text). Juvenile *Sebastes* (Seb) were collected from both East and West Greenland. The *Sebastes mentella* samples (M) from Greenland waters were collected both on surveys (R) and by commercial (C) vessels over 2 years. *Q* refers to sampling zones around Greenland (cf. Figure 1). ID = sample ID, *N *= sample sizes, and NA = data not available. Samples 5, 6, and 7 represent reference samples of *S. mentella* included from the EU REDFISH project

Species	ID	Code	Location	Lat/Long (mean)	Time	*N*	Depth (m)	Avg. length (cm)	Female (%)	Adult (%)
*S. mentella*	1	M‐Nor 1	Northeast Arctic	72.18/10.25	October, 2006	91	340	34	56	100
	2	M‐Nor 2		66.93/8.13	2006, 2009^a^	155	445	34	59	100
	3	M‐Nor 3		67/8.09	March, 2009	76	508	38	83	100
	4	M‐Nor 4		69.38/15.14	November, 2011	91	575	38	60	100
	5	M‐Oc	Irminger Sea	60.41/−39.01	1995, 2001^a^	80	240	37	36	100
	6	M‐Deep		62.55/−27.01	2001	73	845	43	35	100
	7	M‐ICL	Icelandic Shelf	63.28/−26.17	October, 2001	59	651	37	50	92
	8	MC11Q2Q3	East Greenland	64.24/−35.14	March, 2011	137	372	36	56	64
	9	MC11Q2		64.25/−35.16	March, 2011	108	367	37	58	100
	10	MR11Q1Q2		65.95/−33.16	August, 2011	80	348	31	48	31
	11	MR11Q3		63.97/−36.3	August, 2011	69	462	32	38	71
	12	MR11Q5		62.2/−40.65	August, 2011	49	430	31	45	61
	13	MR11Q5Q6		61.15/−41.66	August, 2011	48	452	30	42	56
	14	MU11		NA	November, 2011	26	NA	36	64	76
	15	M1C12Q2		64.57/−35.08	May, 2012	49	375	34	45	99
	16	M2C12Q2		64.52/−35.18	February, 2012	83	375	37	NA	100
	17	M3C12Q2		64.40/−35.23	April, 2012	96	375	35	34	100
	18	MC12Q3		64.33/−35.33	February, 2012	45	375	37	NA	100
	19	MSeb12WGL	West Greenland	68.39/−58.36	June, 2012	91	325	13	NA	NA
	20	MR12Q2_6	East Greenland	59.60/−43.85	June, 2012	93	227	32	48	28
	21	MSeb12Q2		65.46/−30.39	August, 2012	92	561	12	NA	NA
	22	MR12Q6		61.04/−41.65	August, 2012	28	437	33	NA	98
*S. mentella*	23	MR12WGL	West Greenland	68.09/−56.79	June, 2012	89	478	25	NA	33
	24	MR12Q3	East Greenland	64.28/−37.39	August, 2012	89	580	36	47	69
	25	MR12Q4		64.15/−36.81	August, 2012	81	471	29	60	84
	26	MR12Q5		62.19/−40.67	August, 2012	80	448	31	50	89
	27	M‐FC	Flemish Cap	48/−45.16	July, 2001	95	495	28	55	35
*Sebastes norvegicus*	28	Nor‐Nor	Northeast Arctic	69.19/15.08	October, 2001	41	258	39	15	100
	29	Nor‐GL A	East Greenland	64.26/−35.15	February, 2011	108	365	38	58	87
	30	Nor‐GL B		62.2/−40.65	August, 2011	70	239	28	44	29
	31	Nor‐WGL	West Greenland	69.28/−53.1	June, 2012	49	521	53	65	100
	32	Nor‐Giant	East Greenland	61/−29	August, 1996	17	704	79	82	100
*Sebastes viviparus*	33	VV‐Ice	Iceland	64.1/−13.47	March, 2001	53	174	18	47	45
	34	VV‐Nor	Northeast Arctic	70.5/20.52	1992, 2001^a^	26	136	20	NA	100
*Sebastes fasciatus*	35	Fasc	Flemish Cap	45.79/−47.16	October, 2001	45	294	21	62	36

a Samples collected in 2 years. No temporal genetic differences between samples observed.

### 2.2 Microsatellite genotyping

DNA was isolated from ethanol‐preserved gill tissue using the E‐Z 96 Tissue DNA kit according to the manufacturer's instructions (Omega Bio‐Tek, Inc, Norcross, GA, USA). We analyzed a total of 13 microsatellite loci: Seb09, Seb25, Seb31, Seb33, Seb45, Smen05 (Roques, Pallotta, Sevigny, & Bernatchez, 1999), Sal1, Sal3, Sal4 (Miller, Schulze, & Withler, 2000), Smen10 (Stefánsson et al., 2009), and Spi4, Spi6, Spi10 (Gomez‐Uchida, Hoffman, Ardren, & Banks, 2003), arranged in three multiplexes (Table S1). PCR was performed in 2 μl volume comprising 1× Qiagen Multiplex Master Mix (Qiagen, Hilden, Germany), 0.1–1.0 μm primer, and 15–25 ng DNA. The 5′ end on the forward primer was labeled with a fluorescent dye by the manufacturer (Applied Biosystems, Foster City, CA, USA). The GeneAmp 9700 (Applied Biosystems) thermal cycler was used for the amplification, with a PCR profile consisting of an initial denaturation step of 95°C for 15 min followed by 25 cycles of 95°C for 30 s, 56°C for 90 s, and 72°C for 60 s, ending with 60°C for 45 min. The PCR products were size‐separated and genotyped using an ABI 3130 XL automated sequencer (Applied Biosystems) and the GeneMapper 4.0 software (Applied Biosystems). Micro‐Checker (Van Oosterhout, Hutchinson, Wills, & Shipley, 2004) was used to detect possible scoring errors or null alleles in the data. Three of the loci (Spi4, Spi6, and Seb31) were not successfully genotyped for some of the samples of *S. norvegicus* and *S. viviparus*, likely due to poor DNA quality. Eventually those loci were omitted from the analyses of introgression.

### 2.3 Descriptive statistics

We used FSTAT(Goudet, 1995) to calculate the number of alleles and gene diversities for different loci. Allelic richness was estimated for a minimum sample size of 17 individuals in ADZE 1.0 (Szpiech, Jakobsson, & Rosenberg, 2008). Pairwise *F*_ST_ values (Weir & Cockerham, 1984) were estimated in Arlequin 3.5 (Excoffier & Lischer, 2010). Deviations from linkage (LD) and Hardy–Weinberg equilibria were tested in Genepop 4.2 (Rousset, 2008) by Fisher's exact test with the implemented Markov chain Monte Carlo (MCMC) method (dememorization 10,000, batches 1,000, iterations 10,000). Hierarchical analysis of molecular variance (AMOVA) was performed in Arlequin for the *S. mentella* data with three‐group configuration after Bayesian clustering (as described below). False discovery rate control (FDR, Benjamini & Yekutieli, 2001) was applied to avoid type I error, while not losing much power, when multiple comparisons were involved. Genetic diversities, deviations from Hardy–Weinberg and linkage equilibria were also estimated within detected clusters (as described below) to verify pure and admixed fish. For these estimations, comparable sample sizes of fish were selected randomly to represent the pure and admixed clusters.

### 2.4 Population cluster and individual admixture analyses

Population cluster analysis was applied for the 27 samples of *S. mentella* (cf. Table 1; ID: 1–27, *N *=* *2,153) genotyped with 13 microsatellites to detect genetic structuring within the species. To estimate the magnitude of introgression, individual admixture analysis was performed for the total dataset consisting of all 35 samples (*N *=* *2,562) genotyped for ten loci (see rationale above). A Bayesian approach, as implemented in STRUCTURE (Pritchard, Stephens, & Donnelly, 2000), was used for clustering of genotypes and estimation of individual admixture proportion in an effort to identify possible hybrids (burn‐in = 350,000, MCMC = 500,000, replication = 10 for each K). The method clusters individuals to minimize Hardy–Weinberg and gametic phase disequilibria between loci within groups. STRUCTURE may predict fewer than the actual number of clusters in a dataset with hierarchical structure (Kalinowski, 2010), so we ran the program using all samples followed by a cluster by cluster (hierarchical) approach. The Evanno method (Evanno, Regnaut, & Goudet, 2005) as implemented in STRUCTURE HARVESTER (Earl & vonHoldt, 2012) was used to estimate the number of clusters in each dataset.

The incidence of hybridization was further quantified by additional STRUCTURE analyses in clusters that indicated hybridization. To estimate the individual admixture proportions (*Q* = genome ancestry fraction) in STRUCTURE, an admixture model was used. Furthermore, a correlated allele frequency model was applied as the differentiation among the clusters was low (as suggested by Nielsen et al., 2003).

### 2.5 Identification of hybrids

Hybridization of *Sebastes* in waters close to Greenland (Greenland, Irminger Sea, and Iceland) was compared with hybridization in the Northeast Arctic (Norwegian), and Northwest Atlantic waters (Flemish cap) following the approach of Nielsen et al. (2003). An individual was initially identified as possible hybrid if at least 10% of its genome (*Q*) originated from other groups (see Randi, 2008). The most pure parental individuals (*Q* ≥ 0.90) were used as base population for generating *in silico* “pure” individuals using the program HYBRIDLAB (Nielsen, Bach, & Kotlicki, 2006). Simple mechanical mixing was simulated by generating pure parental genotypes (i.e., randomly drawing alleles from the allele frequency distribution of the “pure population”) equal to the numbers of individuals of the estimated clusters. A hybrid swarm (i.e., random mating between species/populations) was generated by random drawing of alleles from the observed allele frequency distribution of the combined species/populations. Differences between these simulated and empirical individual admixture proportions were tested with a Kolmogorov–Smirnov two‐sample (K‐S) test. Potential simulation bias caused by differences in individual admixture proportions between the observed and simulated pure individuals was tested by comparing individual admixture proportions of 20 randomly chosen “pure” individuals of each cluster (i.e., total 40) with 40 “simulated pure” individuals. As *S. norvegicus* could not be sampled from the Northwest Atlantic (Flemish Cap), Norwegian *S. norvegicus* was used as baseline sample for identification of hybrids. Hence, hybrids were investigated only in *S. mentella* and *S. fasciatus* samples for the Northwest Atlantic. The same approach was used for identification of hybrids with *S. viviparus* in Greenland waters as the species has not been identified west of Iceland (Johansen, 2003).

### 2.6 Isolation with migration

The isolation‐with‐migration (IM) model as implemented in IMa2 (Hey, 2009) was applied to estimate introgression and population demographic parameters for the cluster pairs from Greenland waters. The analyses were conducted only for clusters in which hybridization had been indicated through earlier Bayesian clustering analyses (i.e., within *S. mentella* clusters, and across *S. mentella* and *S. norvegicus* clusters). A total of 45 fish within each cluster were randomly selected from the relevant samples. Rather than running all of the clusters together, pairwise cluster comparisons were chosen to reduce computational time. The IM model assumes random mating, free recombination among loci, and no recombination within loci. The MCMC‐based method of the program uses sampling of gene genealogies to estimate posterior probability densities of demographic parameters scaled by mutation rate per generation per year (μ). A stepwise mutation (SM) model was applied for the ten microsatellite loci.

A few preliminary analyses were performed with wider priors. The estimated effective sample size values of parameter *t* (time since divergence), swapping rates, autocorrelation value, trend‐line plots were evaluated. After a burn‐in of 500,000 steps, 2,000,000 more steps were run to save 20,000 genealogies for each pair of clusters. To ensure convergence for the estimation, 150 chains were used for all of runs with high heating schemes (“−ha0.975 −hb0.75”) so that the updates for the chains are accepted at higher rates. Introgression/bidirectional migration rates (m1 and m2) were estimated as effective number of migrants per generation (2*NM* = 4*N*_e_μ*M/μ/2, independent of mutation rate), population size parameters (*Θ *= 4*N*_e_μ, *N*_e_ = effective population size), and time since divergence in generations (*t*μ) without converting for a given mutation rate, and generation time.

## 3 Results

### 3.1 Descriptive statistics

The number of alleles per locus ranged from 13 to 69, and mean allelic richness (*N *=* *17) varied between 3.51 and 20.49. Gene diversities per locus within samples were between 0.14 and 0.969 (mean = 0.77). Significant heterozygote deficits were observed for all loci (most frequently in Greenland waters, Table S2). Nineteen of 35 samples deviated significantly from Hardy–Weinberg expectation before FDR control, and 18 of them remained significant after FDR (Table S2). For the 35 *Sebastes* samples, 162 of 1,575 pairwise tests (10.29%) showed significant deviations from linkage equilibrium. LD was found for fish from all four species (between one and ten significant pairwise comparisons between loci). However, only 21 LD tests remained significant after FDR control. For the 27 *S. mentella* samples, 212 of 2106 tests for linkage equilibrium (approx. 10%) deviated significantly from expected values. After FDR, 57 of those tests remained significant without any spatial pattern. No evidence for null alleles or short allele dominance was detected by Micro‐Checker.

### 3.2 Pattern of genetic differentiation

**Figure 2:**
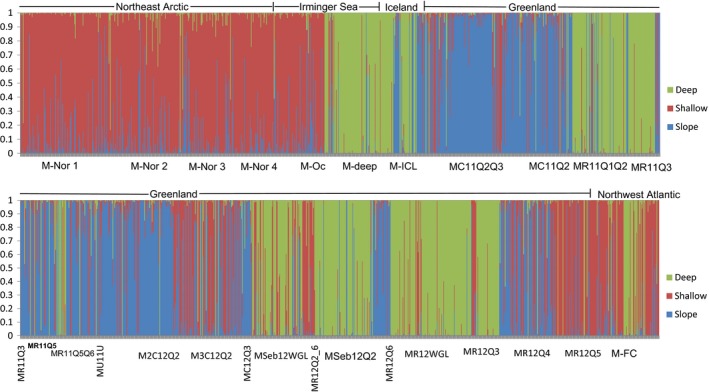
Column charts illustrating individual cluster ancestries obtained from the STRUCTURE analysis (Pritchard et al., 2000) using 13 microsatellites. Three clusters were predicted for the 27 *Sebastes mentella* samples (cf. Table 1). Sample names are provided at the *X*‐axis, while genome ancestry fractions (*Q*) are given at the *Y*‐axis

For the *S. mentella* population structure analysis (27 samples), an initial investigation identified 138 redfish as *S. norvegicus*, which were removed from the subsequent analyses. As no differentiation was observed between temporal samples from two different *S. mentella* locations (Table 1: sample ID 2 and 5), samples were pooled. The subsequent STRUCTURE analyses of the 27 *S. mentella* samples suggested three clusters (Figure 2 and Figure S1). In many cases, individuals from one sample assigned to different clusters. The clusters were sorted according to the occurrence of the reference samples as “shallow (sample 5)/deep (sample 6)/slope (sample 7).” The “shallow” cluster was the largest, including 80% of the “shallow” sampling group fish (M‐Oc, Figure 2) plus individuals from all other *S. mentella* samples (depth = 375 to 575 m). The “deep” cluster consisted of 89% fish from the “deep” sampling group (depth = 830–850 m), fish from East and West Greenland waters (no depth record) in addition to individuals from the Icelandic Shelf (*N* = 40, 612–784 m), Northwest Atlantic (*N* = 26, 450–711 m), Irminger Sea “shallow” (M‐Oc: *N* = 7, 451–735 m), and Northeast Arctic (*N* = 6, 508–575 m) waters. In the “slope” cluster, most individuals (89%) were from East Greenland waters sampled on the shelf (227–350 m). Approximately 29% (*N* = 17) of the “Icelandic Shelf” sample clustered with the “slope” (Figure 2). “Shallow” and “deep” clusters from Greenland waters were distributed in both East and West Greenland, whereas the “slope” cluster was only found in the east. The juvenile sample from East Greenland (MSeb12Q2, depth = 561 m) consisted mainly of the “deep” group fish, with a few individuals from the “slope” group. The two juvenile samples from West Greenland (MSeb12WGL and MR12WGL) were dominated by the “deep” group fish, with few individuals from the “shallow” group. No major seasonal shifts in pattern of the distribution of clusters were observed. However, spring samples of adult fish from commercial vessels were found to be dominated by fish from the “slope” cluster.

**Figure 3:**
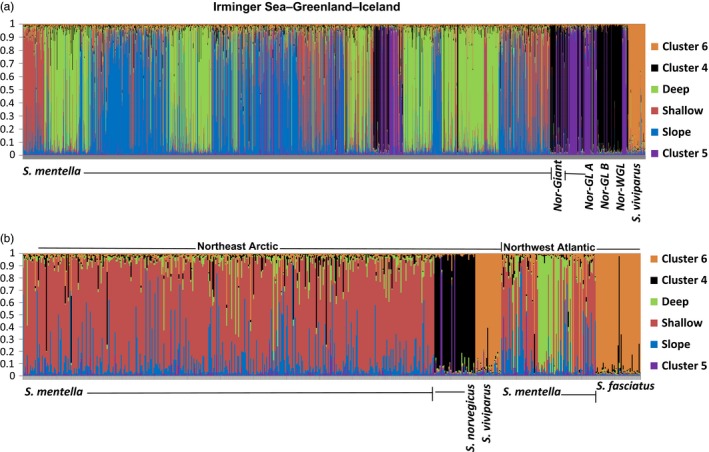
Six genetic clusters (as indicated by different colors) predicted by STRUCTURE for the *Sebastes* spp. samples. Two clusters within the *Sebastes norvegicus* samples are shown in black and purple, and the samples for *Sebastes viviparus* and *Sebastes fasciatus* were clustered together (in orange). Samples have been sorted as per the geographic locations: (a) Irminger Sea–Greenland–Iceland, (b) Northeast Arctic, and Northwest Atlantic

When the total dataset (i.e., 35 samples) was considered, six clusters were identified by STRUCTURE (Figure 3 and Figure S2). Three of the clusters identified within the *S. mentella* samples were consistent with the results described above. Two clusters were detected within the *S. norvegicus* samples, while *S. fasciatus* and *S. viviparus* samples were placed into a single cluster. However, with the application of hierarchical cluster approach, three clusters were detected within the *S. norvegicus* samples and *S. fasciatus* and *S. viviparus* samples were separated into two distinct clusters. For the *S. norvegicus* samples, three clusters were designated as “Norvegicus‐A,” “Norvegicus‐B,” and “Giants” (described in Saha et al., 2016). Mechanical mixing, as consistent with the observed deviations from the Hardy–Weinberg equilibrium, was therefore apparent in multiple samples. As no introgression was associated with the “Giants” cluster, the cluster was not used in the downstream analyses. Evidence of hybridization was observed for almost all possible cluster pairs (Figure 3, see details below).

The three‐group configuration (“shallow–deep–slope”) of *S. mentella* samples predicted by Bayesian approaches was supported by the AMOVA (*F*_CT_ = 0.03, *P* = .000). A much lower, but still significant proportion of the genetic variance could be ascribed to differentiation among samples within groups (*F*_SC_ = 0.002, *P* = .000). The *S. mentella* “shallow” group from Northwest Atlantic (FC) and Northeast Arctic (Nor) were significantly differentiated from each other, and from the Irminger Sea “shallow” group plus “shallow” group samples (except two) from Greenland waters (Table 3). In many of the cases, the “deep” groups identified from the Northwest Atlantic, Irminger Sea, Iceland, and Greenland waters were significantly differentiated from one another (Table 4). Finally, the “slope” group identified from the Northeast Arctic waters was significantly differentiated from Greenland waters (Table 5).

### 3.3 Genetic diversity, Hardy–Weinberg and linkage equilibria within clusters

Genetic diversities, Hardy–Weinberg and linkage equilibria were estimated within the clusters with designated pure and hybrid (*Q* > 10% from other genomes) individuals identified through the admixture analyses. Clusters were formed with comparable numbers of fish (Table S3) to obtain similar statistical power. The cluster of pure *S. fasciatus* (Table S3: Fasciatus) showed significant heterozygote deficiency, only before FDR control. All the admixed clusters except for *S. mentella* and *S. fasciatus*, and the “deep” and “shallow” groups of *S. mentella* were out of Hardy–Weinberg equilibrium. However, none of them remained significant except the admixed cluster of *S. mentella* and *S. viviparus* (“Shallow” × “Viviparus”) after FDR. The admixed clusters had higher gene diversities than one or both of the parental clusters (except “deep–shallow”). A total of 59 (6.39%) tests for deviations from linkage equilibrium within clusters were significant, with more deviations observed in the admixed clusters (eight in Mentella–Fasciatus, six in Norvegicus‐A–B and five in “deep–shallow”).

### 3.4 Introgressive hybridization in the genus *Sebastes*

STRUCTURE revealed admixed individuals for all geographically co‐occurring clusters. Within Greenland waters, we found the highest rate of hybridization among the three *S. mentella* clusters. There were also admixed individuals between *S. mentella* and *S. norvegicus*, and *S. mentella* and *S. viviparus*. Patterns of individual admixture proportions were intermediate between a scenario of mechanical mixing and that of a hybrid swarm (see Figures S3‐S7). No significant simulation bias was observed (Figure S17). Interspecific hybridization appeared to be most prevalent in the Northeast Arctic waters. In these waters, admixed individuals were observed both for *S. mentella–S. norvegicus* and *S. mentella*–*S. viviparus* (Figures S8 and S9). No significant admixture between *S. norvegicus* and *S. viviparus* was detected (Figure S10). In the Northwest Atlantic, hybrid genotypes were observed for *S. mentella–S. norvegicus*,* S. mentella*–*S. fasciatus*, and *S. norvegicus–S. fasciatus* (Figures S11‐S16), but none of the cases conformed to a hybrid swarm scenario.

**Figure 4:**
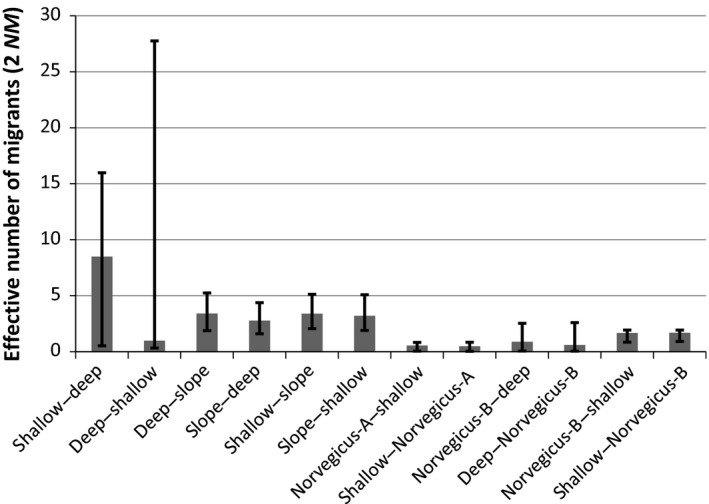
Bidirectional introgression rates for different clusters of *Sebastes* in Greenland waters. The extents of introgression are expressed as effective number of migrants per generation (2*NM*). For the values with highest posterior probabilities, 95% confidence intervals are provided with bars

In Greenland waters, evidence of significant introgression was observed in all cluster pairs studied using the IM model (Figure 4). The highest introgression, which was also the most asymmetric, was observed between the clusters of “shallow” and “deep” *S. mentella*. Hybridization between the “shallow–slope” pair was just higher than for the “deep–slope” pair. In contrast, symmetric patterns of introgression of much lower levels were estimated between Norvegicus‐A–”shallow” Mentella, Norvegicus‐B–”shallow” Mentella, and Norvegicus‐B–”deep” Mentella.

**Figure 5:**
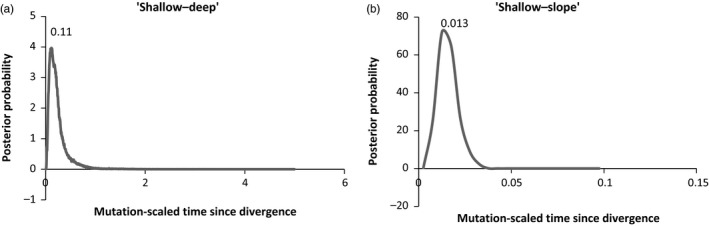
Posterior probability plots of divergence times for the clusters of *S. mentella* complex in Greenland waters. The estimated mutation‐scaled time since divergences in generations (*t*μ) are presented at the apexes of the distributions

**Figure 6:**
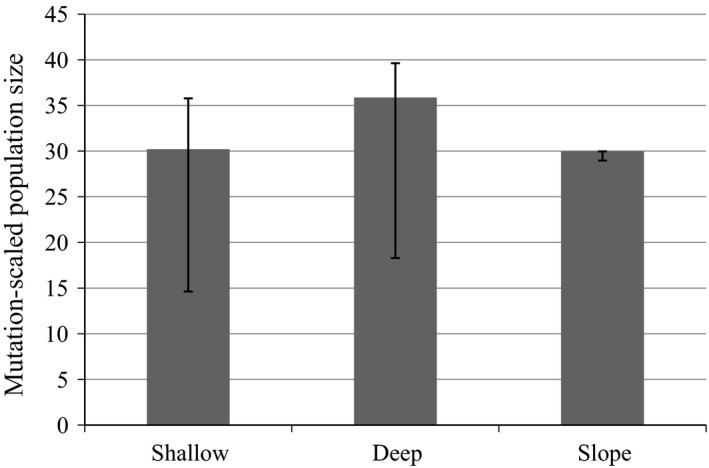
Estimates of the mutation‐scaled population size parameters (*Θ*) for the three groups of *Sebastes mentella* from Greenland waters. For the values with highest posterior probabilities, 95% confidence intervals are provided with bars

The coalescent‐based analysis suggested that the divergence between “shallow” and “deep” groups was more ancient (Figure 5a) than that between the “shallow” and “slope” groups (Figure 5b). The estimates of the population size parameter (*Θ*) were comparable for the three *S. mentella* clusters (Figure 6).

## 4 Discussion

Using an unprecedented large number of target and reference samples from different years, seasons, and life stages, the present investigation provided improved population delineation on a larger geographic scale for the *S. mentella* complex. Our primary results illustrate the presence of three distinct genetic groups within the species across its distribution range. The “shallow” group had a wider distribution than the “deep” and “slope” groups. The occurrence of the “slope” group on the East Greenland Shelf was supported by adult fish collected in both spring and fall, as well as by juveniles. In contrast, the “slope” sample from the Icelandic Shelf was found to be a mixture of fish belonging to both “deep” and “slope” groups. Connectivity in terms of effective number of migrants between the “shallow–slope” pair was found to be greater than connectivity between the “deep–slope” pair. It was also evident that *S. mentella* from the Northeast Arctic and Northwest Atlantic waters are genetically differentiated. Low, but statistically significant, evidence of introgression was observed among the clusters.

### 4.1 Genetic population structure of *Sebastes mentella*

The three groups of *S. mentella* observed in this study are in agreement with earlier findings (for review, see Cadrin et al., 2010). The observation that the “shallow” group includes fish from all 27 geographical samples reveals the widest geographical distribution of this genetic group. Likewise, fish from inside and outside the Irminger Sea were assigned to the “deep” group. These observations are congruent with earlier findings (Shum et al., 2015). The *F*_ST_ estimate between these two distinct genetic groups in the present investigation (*F*_ST_ = 0.03) was higher than that estimated by Stefansson et al. (2009, *F*_ST_ = 0.009) but almost identical to that of Shum et al. (2014, *F*_ST_ = 0.031). Estimates of the demographic history by the IM model revealed that the divergence between these two groups was more ancient (*t*μ = 0.11) than that between “Giants” and “Norvegicus‐B” (*t*μ = 0.06), supporting a deep evolutionary divergence between “shallow” and “deep” groups as suggested by Stefánsson et al. (2009), Shum et al. (2014, 2015).

The “slope” group was mainly observed in East Greenland in catches from the commercial fleets. These samples consisted of adult fish caught during the larval extrusion period (spring) and of juveniles collected in autumn. The finding of juvenile fish is not surprising, because Greenland waters are suggested to be nursery grounds for all *S. mentella* and *S. norvegicus* groups of the region (Anderson, 1984; Magnusson & Johannesson, 1995). However, the presence of adult “slope” individuals suggests that East Greenland waters may also act as an important area of distribution for the adults of this group, which has not previously been recognized.

The finding that 29% of the individuals from the Icelandic Shelf sample were assigned to the “slope” group, but the remaining individuals clustered with “deep,” indicates sympatric occurrence of different groups on the Icelandic Shelf. Previously, it has been indicated that the Icelandic Shelf was the main distributional area for the “slope” group (Cadrin et al., 2010). However, the results presented herein suggest that this distribution extends further into East Greenland waters. At present, the “slope” group *S. mentella* on the Greenland and Iceland Shelf waters are assessed separately by ICES. In light of the results presented here, this should be reconsidered as stock dynamics appear to be linked across continental slopes, and the effect of fishing intensity on either shelf could affect the entire stock unit. A similar scenario has also been described for Atlantic cod where the East Greenland and Iceland slopes are inhabited by the same populations (Therkildsen et al., 2013), and for Greenland halibut in the entire region that is considered as a single unit (ICES 2015b).

The genetic differentiation observed for the “deep–slope” pair was the largest among the three *S. mentella* genetic groups (Table 2). Congruent with this observation, the *F*_ST_ estimates suggested the closest connectivity for the “shallow–slope” pair, which was further supported by the estimates of gene flow (Figure 4) and time of divergence (Figure 5b). In contrast, allozyme studies by Johansen (2003) and Daníelsdóttir et al. (2008) indicated closer relationship for “slope–deep” pair. The discrepancy might be associated with the types of marker applied, because markers can be of varied mode of inheritance, function, and statistical properties. Moreover, the allozyme studies considered each locus separately, rendering low power for estimating connectivity among the genetic groups.

**Table 2:** Pairwise *F*_ST_ values (Weir & Cockerham 1984) among the three groups of *Sebastes mentella* predicted by STRUCTURE (cf. Figure 2)

	*N*	Shallow	Slope
Shallow	786	–	
Slope	634	0.025	
Deep	595	0.030	0.037

The values are significant at *P *=* *.000. *N *= Sample size.

Based on the results from the hierarchical variance analysis (AMOVA), there was evidence of statistically significant genetic variance among samples within groups. This result was also supported by the many significant pairwise *F*_ST_ estimates between samples within groups. Most importantly, the identification of significant differentiation between the Northeast Arctic sample and other samples within the “shallow” group (Table 3) suggests the existence of isolated genetic components in the region. The elaborate sampling facilitated the testing of temporal stability (i.e., samples from 2006, 2009, and 2011) in the occurrence and genetic composition of the Northeast Arctic component. This component was previously identified by Roques, Sevigny, and Bernatchez (2002) and by Johansen (2003), but not by Stefansson et al. (2009). An isolated genetic component of *S. mentella* in the Northeast Arctic is also supported by the finding of a separate larval extrusion and nursery ground in this region (e.g., Cadrin et al., 2010). The finding that *S. mentella* from the Northwest Atlantic were significantly differentiated from other *S. mentella* samples (Tables 3 and 4) supports the idea of a “Western stock” as proposed by Cadrin et al. (2010). The highest substructuring was found within the “deep” group (Tables 3, 4, 5), indicating higher habitat segregation for the group in comparison with “shallow” and “slope.” This observation is consistent with Shum et al. (2015) who hypothesized based on their mtDNA data that female “deep” *S. mentella* may exhibit some degree of philopatry.

**Table 3:** Pairwise *F*_ST_ values (Weir & Cockerham 1984) within *Sebastes mentella* “shallow” group (cf. Figure 2)

	*N*	Greenland commercial		FC	Nor	M‐Oc	Greenland research			
		C11Q2Q3	C12Q2Q3				R11Q3Q5Q6	R12Q2Q4	R12Q3Q5Q6	R12WGL
C11Q2Q3	33	–								
C12Q2Q3	66	0.001								
FC	60	**0.004**	**0.003**							
Nor	377	0.001	0.001	0.004*						
M‐Oc (shallow)	64	−0.001	**0.002**	0.005*	**0.002**					
R11Q3Q5Q6	19	−0.003	−0.001	0.001	−0.001	0.000				
R12Q2Q4	39	−0.003	−0.002	0.001	−0.003	−0.003	−0.006			
R12Q3Q5Q6	75	**0.004**	0.005*	0.007*	0.005*	0.004*	0.003	0.001		
R12WGL	12	0.002	0.007	0.006	0.004	0.003	0.001	−0.003	0.004	
Seb12WGL	27	0.001	−0.001	0.003	0.003	**0.004**	−0.002	−0.002	0.004	0.006

*N *= Sample size. Closely located small samples from Greenland waters were pooled. For the sample codes, Nor = Samples 1–4, M‐Oc = Sample 5 (reference sample “shallow”), FC = Sample 27, Seb12WGL = Sample 19, and R12WGL = Sample 23 (cf. Table 1).

The values in bold are significant at *P* = .05, while asterisks indicate significance after false discovery rate control.

**Table 4:** Pairwise *F*_ST_ values (Weir & Cockerham 1984) within *Sebastes mentella* “deep” group (cf. Figure 2)

	*N*	Greenland commercial		M‐Deep	FC	M‐ICL	Greenland research				
		C11Q2Q3	C12Q2Q3				R11Q1Q2	R11Q3Q5Q6	R12Q3Q5Q6	R12WGL	Seb12Q2
C11Q2Q3	9	–									
C12Q2Q3	9	0.000									
M‐Deep	65	0.005	−0.001								
FC	26	**0.011**	0.004	**0.006**							
M‐ICL	40	0.002	0.000	**0.003**	0.002						
R11Q1Q2	65	0.008	0.005	0.006*	0.003	−0.001					
R11Q3Q5Q6	61	0.009	0.004	0.005*	0.003	−0.001	0.000				
R12Q3Q5Q6	81	0.006	−0.001	0.001	0.001	−0.001	0.000	0.000			
R12WGL	74	0.003	−0.003	**0.002**	**0.004**	−0.001	0.001	0.002	−0.001		
Seb12Q2	80	0.008	0.000	**0.002**	**0.004**	−0.001	0.000	0.002	−0.001	0.000	
Seb12WGL	54	0.004	−0.002	0.001	0.000	0.001	0.001	**0.003**	−0.001	0.000	0.000

*N *= Sample size. Closely located small samples from Greenland waters were pooled. For the sample codes, FC = Sample 27, M‐Deep = Sample 6 (reference sample “deep”), M‐ICL = Sample 7 (reference sample “Icelandic slope”), Seb12WGL = Sample 19, Seb12Q2 = Sample 21, and R12WGL = Sample 23 (cf. Table 1).

Significant at *P *=* *.05 in bold. Asterisks indicate significance after false discovery rate control.

**Table 5:** Pairwise *F*_ST_ values (Weir & Cockerham 1984) within *Sebastes mentella* “slope” group (cf. Figure 2)

	*N*	Greenland commercial		M‐ICL	Nor	M‐Oc	Greenland research			
		C11Q2Q3	C12Q2Q3				R11Q1Q2	R11Q3Q5Q6	R12Q2Q4	R12Q3Q5Q6
C11Q2Q3	219	–								
C12Q2Q3	155	0.000								
M‐ICL	17	0.000	0.000							
Nor	27	0.009*	**0.005**	0.004						
M‐Oc (shallow)	9	0.001	−0.004	−0.003	−0.011					
R11Q1Q2	11	0.001	0.001	0.005	0.018*	−0.001				
R11Q3Q5Q6	85	0.000	0.001	0.004	0.009*	0.001	0.000			
R12Q2Q4	51	0.000	0.001	0.004	0.009*	−0.001	0.001	0.000		
R12Q3Q5Q6	37	0.002	0.003	0.004	0.009*	−0.001	0.005	0.003	0.003	
Seb12Q2	8	0.003	0.005	0.010	**0.016**	0.006	0.003	0.002	−0.009	0.002

*N *= Sample size. Closely located small samples from Greenland waters were pooled. For the sample codes, Nor = Samples 1–4, M‐Oc = Sample 5 (reference sample “shallow”), M‐ICL = Sample 7 (reference sample “Icelandic slope”), and Seb12Q2 = Sample 21 (cf. Table 1).

Significant at *P *=* *.05 in bold. Asterisks indicate significance after false discovery rate control.

Both the “deep” and “slope” groups were mainly concentrated in the central North Atlantic. Nevertheless, a significant number of fish from the Northwest Atlantic assigned to “deep” group, and some fish from Northeast Arctic ascribed to the “slope” group. The “shallow” group was distributed across the North Atlantic with evidence of variance among samples within the group. Such trans‐Atlantic genetic structuring have been indicated for the entire *S. mentella* complex (Cadrin et al., 2010), but is genetically confirmed only for the “shallow” group (Shum et al., 2015), which could be associated with the less extensive sampling in earlier studies. However, trans‐Atlantic patterns of genetic population structure have been observed in other species with comparable genetic structuring such as Atlantic cod (Bradbury et al., 2013; Hemmer‐Hansen et al., 2013). It may be hypothesized that the apparent genetic pattern is possibly associated with separate glacial refugia and trans‐Atlantic gene flow driven by the warm interglacial periods, an explanation in line with Shum et al. (2015), Bradbury et al. (2013), and Hemmer‐Hansen et al. (2013).

### 4.2 Introgression within the genus

Strong evidence of introgression among different clusters of *Sebastes* was found, in terms of deviations from Hardy–Weinberg expectations and linkage equilibrium, which was also consistent with the downstream Bayesian admixture and IM analyses. Although deviations from Hardy–Weinberg expectations and linkage equilibrium only provide qualified support for introgression, an admixed group of individuals should show more extensive deviations than a group of pure individuals (Scribner, 1993), as seen when comparing clusters with pure and admixed individuals (Table S3). The reason why the apparently nonadmixed *S. fasciatus* cluster (Figure 3) still displayed such deviations could be due to the inclusion of undetected hybrids with other clusters not included in the baseline such as *S. fasciatus*–*S. norvegicus*. This was supported by the detection of admixed individuals in the simulation including Norwegian *S. norvegicus* as baseline. In contrast, a few apparently admixed clusters did not display such deviations, which could be associated with the low power of those tests (i.e., as suggested by Nielsen et al., 2003). Another indication of introgression is the elevated levels of polymorphism observed, which is expected for mixed gene pools (e.g., Roques et al., 2001).

The extent of introgression was greater among the three groups of *S. mentella* than among other clusters (Figure 4 and Figures S3‐S16). However, the magnitude observed in our study was less than those reported for *S. mentella–S. norvegicus* (Pampoulie & Daníelsdóttir, 2008), or for *S. mentella*–*S. viviparus* (Artamonova et al., 2013 cf. Figure S7). A low level of introgression within the redfish from these regions has been reported by Saha et al. (2016) and Schmidt (2005). The discrepancies with other studies might be associated with differences in statistical power among different studies. Incomplete or relaxed reproductive barriers among closely related species may provide opportunities for hybridization when in sympatry (Barton & Hewitt, 1989). Speciation within the genus *Sebastes* is a recent event (Briggs, 1995), and thus, these species may have relaxed reproductive barriers allowing hybridization to some extent (e.g., Roques et al., 2001). Nevertheless, *Sebastes* are ovoviviparous and display particular mating behavior during copulation (Helvey, 1982; Kendall, 1991) which implies that large‐scale introgression may require more than simple sympatric existence, as evident in the Gulf of St. Lawrence (Roques et al., 2001).

The pattern of introgression between “shallow” and “deep” groups in Greenland waters appeared asymmetric (Figure 4). Similar observations have been made in other studies of *Sebastes*, and the roles of selection and differential abundance have been considered (Roques et al., 2001; Seeb, 1998). Considering the estimates of population size parameters in Greenland waters (Figure 6), more introgression would be expected from the “deep” group toward the “shallow,” because smaller populations are more likely to be introgressed by more abundant populations over time (Arnold, Hamrick, & Bennett, 1993). However, the opposite was observed. The estimates of the population size were comparable with overlapping confidence intervals. Therefore, other factors than population size, such as selection, may be more likely as drivers of the asymmetric pattern of introgression. The spatial and temporal overlap between the groups during mating may also not be reflected by the population sizes *per se*.

The greatest extent of interspecific hybridization was observed in the Northeast Arctic waters (Figures S8 and S9). The results imply that *S. mentella* (“shallow”) in the Northeast Arctic waters hybridize more frequently with both *S. viviparus* and *S. norvegicus* (“Norvegicus‐B”) than in other areas. One plausible reason for this might be the differential abundance of species. Recent surveys conducted in the Northeast Arctic waters have indicated a much greater abundance of *S. mentella* than *S. norvegicus* (Drevetnyak, Nedreaas, & Planque, 2011; ICES 2015c). Data on the *S. viviparus* stock size are sparse. But, the observed introgression in *S. mentella* might be one of the factors influencing allele frequency differences in the samples from the Northeast Arctic, an explanation in agreement with the Northwest component of this species (Roques et al., 2001).

Heterozygote deficiencies were apparent in some loci for several samples, consistent with observations using some of these loci in other studies (e.g., Pampoulie & Daníelsdóttir, 2008; Roques et al., 2001; Schmidt, 2005). No null alleles or short allele dominances were detected. Furthermore, sampling was not always in the mating area or during the mating period, and morphological identification of these species is uncertain (Barsukov et al., 1984). Therefore, mechanical mixing was considered as likely explanation of the observed heterozygote deficiencies.

Although the present investigation provides evidence of introgression between the identified *Sebastes* gene pools in sympatry, it may be difficult in some cases to distinguish the signal of introgression from noise. STRUCTURE has a tendency to misclassify “pure” individuals as “hybrids” when low differentiation is observed between the gene pools (Bohling, Adams, & Waits, 2013). As discussed earlier, introgression was supported by other genetic analyses such as IM results and the magnitude of introgression observed in our study is less than those reported in other investigations (e.g., Artamonova et al., 2013; Stefánsson et al., 2009). It is evident that hybridization was not of similar magnitude throughout the North Atlantic. For instance, interspecific hybridization appeared to be larger in Norwegian waters, whereas very low magnitude of introgression was observed in other areas (e.g., Figures S6, S7, S10 and S12–S14). Inclusion of more loci might have provided more power for the hybridization analyses. However, the number of loci used in our hybridization analyses was larger than that used by Roques et al. (2001) in a study of genus *Sebastes* and Nielsen et al. (2003) on cod.

The generally wide confidence intervals for our demographic estimates may reflect that the data do not allow precise estimation of the demographic history of these species and populations. Only one pair of clusters was considered at a time, thereby ignoring the role of other contemporary clusters (i.e., “ghost populations”). This approach may limit the applicability of the IM method for providing realistic estimates of introgression and demographic parameters. However, no severe bias has been observed in other comparable studies (Chan et al., 2013; Jacobsen & Omland, 2012; Won & Hey, 2005), so possible bias in our findings is believed to be minor and have little impact on our main conclusions.

The SM model is only option for IM analyses of microsatellite data, but microsatellites probably do not follow this model. Strasburg and Rieseberg (2009) analyzed sequence data and compared infinite sites and HKY substitution models for IM analyses, which may be similar between SM and infinite site model for microsatellite data. They report limited bias in IM results, with the exception of ancestral population size (which is not the objective of our study). Moreover, if the mutation model is violated, it is violated in all analyses. We are interested in relative magnitude of gene flow and time since divergence, and even if absolute values may be affected by such violations of assumptions, the relative comparison is likely to produce valid results.

Lack of knowledge on stock identity and scientific discrepancy in perception of stock entities of *S. mentella* especially in the Irminger Sea have caused failure of an efficient management of the stocks for the latest decade. The poor knowledge of biological parameters for the species and of stock identity have resulted in uncertain advice over the past (ICES 2015a). This lack of robustness in advice has caused the main client, the North East Atlantic Fisheries Commission (NEAFC), to fail reaching consensus on common management for the pelagic stocks of *S. mentella*, and catches have consequently exceeded biological advice by more than three times due to autonomous quota setting by each fishing nation (ICES 2015a). The further clarification of stock identity for *S. mentella* in the present study is anticipated to lead to a more robust advice which again likely will ensure a sustainable fishery by a common fishery management.

## 5 Conclusions

We provide the first genetic investigation of *S. mentella* throughout its range, including Greenland waters. The identification of all three *S. mentella* groups in Greenland waters supports the interpretation of this region as a nursery area and population mixing zone. Fish from the “shallow” group were identified across the North Atlantic, but divided into three populations: Northeast Arctic, Irminger Sea/Greenland, and Northwest Atlantic. Therefore, we clarify the genetic stock identity of *S. mentella* from the Northeast Arctic, which was previously disputed. The “deep” group *S. mentella* were identified in the Irminger Sea and Greenland waters, but some were also found in the Northwest Atlantic. The “slope” fish from the Icelandic Shelf and Greenland waters were not genetically differentiated, which suggests genetic connectivity of “slope” fish in East Greenland–Iceland Shelves. In Greenland waters, the “slope” group was the main target for the commercial fleets in spring. Although genetic heterogeneity was evident, low‐to‐moderate extent of gene flow was observed across the North Atlantic, implying incomplete reproductive isolation for *Sebastes* clusters possibly due to their close evolutionary relationship. The genetic differentiation between *S. mentella* groups were less than that between the other *Sebastes* clusters. Our findings mostly support the existing management practice of *S. mentella* throughout the North Atlantic (Cadrin et al., 2010, 2011), which strengthen the population genetic basis of stock boundaries. The consideration of all stock identities for these species into the management practices will help to manage sustainable utilization of this important fishery resource.

## Data Archiving

Data available from the Dryad Digital Repository: http://dx.doi.org/10.5061/dryad.94d1s.

## Conflict of Interest

No conflict of interest declared.
