# Supplementary material for: Geographic extent of introgression in Sebastes mentella and its effect on genetic population structure
Source: Evol Appl. 2016 Oct 22;10(1):77–90. doi: 10.1111/eva.12429 (PMC5192944; doi:10.1111/eva.12429)
Supplement: Supplementary file 1 [file EVA-10-77-s001.doc]

**Supplementary data**

**Table S1** Overview of the microsatellite loci used in the study.

| **Locus** | **Repeat units** | **Multiplex** | **[Primer] µM** | **References** |
| --- | --- | --- | --- | --- |
| Sal1 | 4 | 1 | 1.00 | Miller et al. (2000) |
| Sal3 | 5 | 1 | 1.00 |  |
| Sal4 | 4 | 1 | 0.10 |  |
| Smen5 | 4 | 1 | 1.00 | Roques et al. (1999) |
| Smen10 | 4 | 2 | 0.75 | [Stefánsson et al. (2009b](#_ENREF_47)) |
| Spi4 | 2 | 2 | 1.00 | [Gomez-Uchida et al. (2003](#_ENREF_16)) |
| Spi10 | 4 | 2 | 0.25 |  |
| Spi6 | 4 | 2 | 1.00 |  |
| SEB09 | 2 | 3 | 0.50 | Roques et al. (1999) |
| SEB25 | 2 | 3 | 0.50 |  |
| SEB45 | 2 | 3 | 1.00 |  |
| SEB31 | 2 | 3 | 0.75 |  |
| SEB33 | 2 | 3 | 1.00 |  |

**Table S2** Sample names and basic data for the studied microsatellite loci. *N*_A_ = number of alleles, *A*_R_ = corrected allelic richness, *H*_O_ and *H*_E_ = observed and expected heterozygosity, and *F*_IS_ = inbreeding coefficient . Bold *F*_IS_ values are significant at *P* = 0.05 (cf. Table 1). Asterisks indicate significance after FDR control. FDR is only applied to overall *F*_IS_ values.

| **Name** | **Parameters** | **Sal1** | **Sal3** | **Sal4** | **Smen05** | **Smen10** | **Spi10** | **Seb09** | **Seb25** | **Seb33** | **Seb45** | **Spi4** | **Spi6** | **Seb31** | **Overall** |
| --- | --- | --- | --- | --- | --- | --- | --- | --- | --- | --- | --- | --- | --- | --- | --- |
|  | ***N*_A_** | 19 | 7 | 4 | 14 | 9 | 14 | 10 | 13 | 34 | 26 | 13 | 15 | 14 |  |
|  | ***A*_R_** | 12.94 | 6.16 | 3.19 | 9.54 | 6.80 | 9.24 | 7.81 | 8.93 | 20.41 | 12.94 | 9.74 | 9.71 | 8.82 |  |
| M-Nor 1 | ***H*_O_** | 0.89 | 0.73 | 0.42 | 0.79 | 0.75 | 0.64 | 0.63 | 0.87 | 0.99 | 0.86 | 0.88 | 0.82 | 0.81 |  |
|  | ***H*_E_** | 0.90 | 0.72 | 0.38 | 0.82 | 0.78 | 0.68 | 0.65 | 0.82 | 0.96 | 0.87 | 0.87 | 0.86 | 0.76 |  |
|  | ***F*_IS_** | 0.01 | -0.01 | -0.11 | 0.04 | 0.04 | 0.07 | 0.04 | -0.06 | -0.03 | 0.02 | -0.01 | 0.04 | -0.07 | 0.001 |
|  |  |  |  |  |  |  |  |  |  |  |  |  |  |  |  |
|  | ***N*_A_** | 18 | 6 | 5 | 12 | 10 | 14 | 15 | 16 | 35 | 26 | 15 | 13 | 17 |  |
|  | ***A*_R_** | 11.74 | 5.46 | 3.28 | 9.21 | 7.11 | 8.76 | 8.95 | 9.47 | 19.96 | 14.30 | 9.98 | 8.56 | 9.62 |  |
| M-Nor 2 | ***H*_O_** | 0.94 | 0.69 | 0.31 | 0.83 | 0.78 | 0.66 | 0.68 | 0.82 | 0.92 | 0.89 | 0.86 | 0.83 | 0.83 |  |
|  | ***H*_E_** | 0.88 | 0.70 | 0.31 | 0.84 | 0.79 | 0.68 | 0.69 | 0.83 | 0.95 | 0.90 | 0.86 | 0.85 | 0.79 |  |
|  | ***F*_IS_** | -0.06 | 0.01 | 0.00 | 0.00 | 0.02 | 0.02 | 0.02 | 0.01 | 0.03 | 0.01 | 0.01 | 0.02 | -0.04 | 0.003 |
|  |  |  |  |  |  |  |  |  |  |  |  |  |  |  |  |
|  | ***N*_A_** | 17 | 6 | 3 | 14 | 8 | 15 | 13 | 15 | 32 | 24 | 14 | 17 | 16 |  |
|  | ***A*_R_** | 12.19 | 5.46 | 2.97 | 9.96 | 6.48 | 10.40 | 8.80 | 9.35 | 19.80 | 13.55 | 9.66 | 10.37 | 9.84 |  |
| M-Nor 3 | ***H*_O_** | 0.82 | 0.66 | 0.32 | 0.84 | 0.75 | 0.78 | 0.74 | 0.82 | 0.98 | 0.92 | 0.92 | 0.79 | 0.74 |  |
|  | ***H*_E_** | 0.88 | 0.68 | 0.33 | 0.84 | 0.77 | 0.83 | 0.68 | 0.79 | 0.95 | 0.88 | 0.87 | 0.86 | 0.78 |  |
|  | ***F*_IS_** | **0.08** | 0.04 | 0.04 | 0.01 | 0.03 | 0.06 | -0.08 | -0.03 | -0.03 | -0.05 | -0.06 | 0.08 | 0.05 | 0.009 |
|  |  |  |  |  |  |  |  |  |  |  |  |  |  |  |  |
|  | ***N*_A_** | 16 | 7 | 3 | 12 | 9 | 13 | 12 | 14 | 33 | 21 | 13 | 13 | 13 |  |
|  | ***A*_R_** | 12.30 | 5.52 | 2.97 | 9.51 | 6.53 | 8.06 | 8.31 | 9.12 | 20.95 | 13.07 | 10.04 | 9.74 | 8.69 |  |
| M-Nor 4 | ***H*_O_** | 0.89 | 0.64 | 0.43 | 0.88 | 0.73 | 0.72 | 0.76 | 0.84 | 0.96 | 0.92 | 0.91 | 0.83 | 0.64 |  |
|  | ***H*_E_** | 0.90 | 0.67 | 0.42 | 0.84 | 0.79 | 0.68 | 0.67 | 0.83 | 0.96 | 0.88 | 0.86 | 0.87 | 0.69 |  |
|  | ***F*_IS_** | 0.01 | 0.05 | -0.01 | -0.05 | 0.07 | -0.05 | -0.14 | -0.01 | 0.00 | -0.05 | -0.05 | 0.05 | 0.08 | -0.008 |
|  |  |  |  |  |  |  |  |  |  |  |  |  |  |  |  |
| **Table S2** | **Contd.** |  |  |  |  |  |  |  |  |  |  |  |  |  |  |
| **Name** | **Parameters** | **Sal1** | **Sal3** | **Sal4** | **Smen05** | **Smen10** | **Spi10** | **Seb09** | **Seb25** | **Seb33** | **Seb45** | **Spi4** | **Spi6** | **Seb31** | **Overall** |
|  | ***N*_A_** | 16 | 6 | 3 | 12 | 10 | 20 | 13 | 14 | 31 | 21 | 12 | 14 | 16 |  |
|  | ***A*_R_** | 11.89 | 5.31 | 2.79 | 9.58 | 7.60 | 10.36 | 8.16 | 8.55 | 19.64 | 13.50 | 9.56 | 8.71 | 10.07 |  |
| M-Oc | ***H*_O_** | 0.83 | 0.70 | 0.21 | 0.84 | 0.82 | 0.79 | 0.78 | 0.85 | 0.92 | 0.91 | 0.89 | 0.93 | 0.74 |  |
|  | ***H*_E_** | 0.89 | 0.68 | 0.23 | 0.85 | 0.79 | 0.73 | 0.76 | 0.82 | 0.95 | 0.88 | 0.87 | 0.84 | 0.79 |  |
|  | ***F*_IS_** | 0.06 | -0.02 | 0.06 | 0.02 | -0.04 | -0.07 | -0.02 | -0.04 | 0.03 | -0.03 | -0.02 | -0.10 | 0.07 | -0.011 |
|  |  |  |  |  |  |  |  |  |  |  |  |  |  |  |  |
|  | ***N*_A_** | 17 | 7 | 3 | 11 | 11 | 34 | 11 | 23 | 35 | 20 | 15 | 17 | 19 |  |
|  | ***A*_R_** | 13.23 | 4.77 | 2.90 | 8.93 | 8.36 | 19.87 | 8.03 | 13.86 | 21.22 | 13.41 | 9.88 | 12.05 | 13.12 |  |
| M-Deep | ***H*_O_** | 0.92 | 0.58 | 0.26 | 0.75 | 0.83 | 0.96 | 0.85 | 0.81 | 0.96 | 0.90 | 0.84 | 0.90 | 0.93 |  |
|  | ***H*_E_** | 0.91 | 0.56 | 0.24 | 0.85 | 0.85 | 0.95 | 0.83 | 0.84 | 0.96 | 0.91 | 0.84 | 0.90 | 0.90 |  |
|  | ***F*_IS_** | -0.01 | -0.04 | -0.10 | **0.12** | 0.02 | -0.01 | -0.02 | 0.03 | 0.00 | 0.01 | 0.01 | -0.01 | -0.04 | 0.004 |
|  |  |  |  |  |  |  |  |  |  |  |  |  |  |  |  |
|  | ***N*_A_** | 16 | 7 | 3 | 13 | 9 | 29 | 10 | 18 | 27 | 23 | 11 | 16 | 15 |  |
|  | ***A*_R_** | 13.04 | 4.94 | 2.60 | 10.32 | 7.08 | 19.03 | 7.01 | 12.34 | 19.36 | 15.75 | 8.79 | 11.99 | 11.08 |  |
| M-ICL | ***H*_O_** | 0.93 | 0.60 | 0.12 | 0.81 | 0.81 | 0.88 | 0.69 | 0.75 | 0.91 | 0.93 | 0.83 | 0.73 | 0.73 |  |
|  | ***H*_E_** | 0.92 | 0.58 | 0.14 | 0.88 | 0.78 | 0.94 | 0.71 | 0.81 | 0.96 | 0.92 | 0.83 | 0.89 | 0.82 |  |
|  | ***F*_IS_** | -0.01 | -0.04 | 0.18 | 0.08 | -0.05 | **0.07** | 0.02 | 0.08 | 0.04 | -0.02 | 0.00 | **0.19** | **0.12** | 0.005 |
|  |  |  |  |  |  |  |  |  |  |  |  |  |  |  |  |
|  | ***N*_A_** | 14 | 7 | 5 | 14 | 11 | 15 | 11 | 17 | 31 | 26 | 14 | 18 | 18 |  |
|  | ***A*_R_** | 10.69 | 4.59 | 3.41 | 10.01 | 6.97 | 8.78 | 6.99 | 10.68 | 18.16 | 14.73 | 8.18 | 10.62 | 10.65 |  |
| MC11Q2Q3 | ***H*_O_** | 0.89 | 0.70 | 0.22 | 0.72 | 0.62 | 0.74 | 0.72 | 0.82 | 0.89 | 0.92 | 0.79 | 0.75 | 0.80 |  |
|  | ***H*_E_** | 0.87 | 0.67 | 0.22 | 0.84 | 0.66 | 0.81 | 0.72 | 0.85 | 0.95 | 0.91 | 0.80 | 0.86 | 0.78 |  |
|  | ***F*_IS_** | -0.03 | -0.03 | 0.01 | **0.14** | 0.06 | **0.09** | 0.00 | 0.03 | **0.06** | -0.01 | 0.01 | **0.13** | -0.04 | **0.004*** |
|  |  |  |  |  |  |  |  |  |  |  |  |  |  |  |  |
|  | ***N*_A_** | 14 | 6 | 4 | 15 | 10 | 16 | 8 | 18 | 35 | 24 | 11 | 15 | 20 |  |
|  | ***A*_R_** | 10.79 | 4.68 | 3.70 | 10.68 | 6.48 | 9.22 | 6.14 | 10.93 | 18.27 | 14.53 | 7.67 | 9.82 | 11.28 |  |
| MC11Q2 | ***H*_O_** | 0.85 | 0.72 | 0.33 | 0.72 | 0.64 | 0.83 | 0.64 | 0.85 | 0.94 | 0.87 | 0.74 | 0.78 | 0.75 |  |
| **Table S2** | **Contd.** |  |  |  |  |  |  |  |  |  |  |  |  |  |  |
| **Name** | **Parameters** | **Sal1** | **Sal3** | **Sal4** | **Smen05** | **Smen10** | **Spi10** | **Seb09** | **Seb25** | **Seb33** | **Seb45** | **Spi4** | **Spi6** | **Seb31** | **Overall** |
| MC11Q2 | ***H*_E_** | 0.87 | 0.70 | 0.38 | 0.87 | 0.63 | 0.80 | 0.71 | 0.88 | 0.95 | 0.91 | 0.75 | 0.87 | 0.78 |  |
|  | ***F*_IS_** | 0.02 | -0.03 | 0.13 | **0.17** | -0.01 | -0.04 | **0.10** | 0.03 | 0.01 | 0.04 | 0.02 | **0.11** | 0.04 | **0.004*** |
|  |  |  |  |  |  |  |  |  |  |  |  |  |  |  |  |
|  | ***N*_A_** | 18 | 7 | 4 | 13 | 10 | 34 | 10 | 20 | 32 | 24 | 12 | 14 | 20 |  |
|  | ***A*_R_** | 13.86 | 5.02 | 2.65 | 10.32 | 7.73 | 18.10 | 7.83 | 12.99 | 20.45 | 16.25 | 8.83 | 11.14 | 12.15 |  |
| MR11Q1Q2 | ***H*_O_** | 0.89 | 0.59 | 0.11 | 0.82 | 0.81 | 0.90 | 0.70 | 0.87 | 0.92 | 0.86 | 0.89 | 0.74 | 0.76 |  |
|  | ***H*_E_** | 0.93 | 0.59 | 0.11 | 0.87 | 0.84 | 0.93 | 0.74 | 0.87 | 0.96 | 0.93 | 0.85 | 0.88 | 0.86 |  |
|  | ***F*_IS_** | 0.04 | 0.00 | -0.03 | 0.05 | 0.03 | 0.04 | 0.06 | 0.00 | 0.04 | **0.08** | -0.05 | **0.16** | **0.11** | **0.05*** |
|  |  |  |  |  |  |  |  |  |  |  |  |  |  |  |  |
|  | ***N*_A_** | 17 | 6 | 5 | 12 | 8 | 28 | 9 | 19 | 35 | 24 | 13 | 17 | 19 |  |
|  | ***A*_R_** | 13.43 | 4.52 | 3.21 | 9.89 | 7.35 | 17.18 | 7.25 | 11.71 | 20.79 | 15.93 | 8.91 | 12.04 | 12.60 |  |
| MR11Q3 | ***H*_O_** | 0.84 | 0.57 | 0.14 | 0.84 | 0.75 | 0.91 | 0.74 | 0.80 | 0.91 | 0.94 | 0.87 | 0.76 | 0.83 |  |
|  | ***H*_E_** | 0.92 | 0.62 | 0.19 | 0.88 | 0.83 | 0.94 | 0.72 | 0.84 | 0.96 | 0.92 | 0.85 | 0.89 | 0.88 |  |
|  | ***F*_IS_** | **0.09** | 0.08 | **0.24** | 0.05 | 0.09 | 0.02 | -0.02 | 0.05 | 0.05 | -0.03 | -0.02 | **0.14** | 0.07 | **0.05*** |
|  |  |  |  |  |  |  |  |  |  |  |  |  |  |  |  |
|  | ***N*_A_** | 13 | 5 | 4 | 13 | 10 | 17 | 9 | 15 | 26 | 19 | 12 | 12 | 14 |  |
|  | ***A*_R_** | 11.24 | 4.40 | 3.73 | 9.58 | 7.29 | 11.46 | 7.32 | 11.62 | 18.98 | 14.73 | 9.45 | 9.58 | 9.99 |  |
| MR11Q5 | ***H*_O_** | 0.79 | 0.73 | 0.33 | 0.85 | 0.59 | 0.86 | 0.67 | 0.92 | 0.96 | 0.92 | 0.78 | 0.73 | 0.61 |  |
|  | ***H*_E_** | 0.89 | 0.69 | 0.39 | 0.84 | 0.62 | 0.85 | 0.70 | 0.88 | 0.95 | 0.90 | 0.82 | 0.86 | 0.72 |  |
|  | ***F*_IS_** | **0.12** | -0.07 | 0.17 | -0.01 | 0.04 | 0.00 | 0.04 | -0.04 | -0.01 | -0.02 | 0.05 | **0.15** | **0.16** | 0.04 |
|  |  |  |  |  |  |  |  |  |  |  |  |  |  |  |  |
|  | ***N*_A_** | 14 | 6 | 4 | 12 | 8 | 17 | 10 | 16 | 27 | 23 | 12 | 14 | 16 |  |
|  | ***A*_R_** | 12.17 | 4.82 | 3.35 | 10.65 | 6.71 | 11.22 | 7.69 | 11.91 | 18.95 | 15.37 | 9.35 | 10.28 | 12.28 |  |
| MR11Q5Q6 | ***H*_O_** | 0.90 | 0.52 | 0.35 | 0.94 | 0.79 | 0.85 | 0.63 | 0.85 | 0.91 | 0.88 | 0.77 | 0.88 | 0.83 |  |
|  | ***H*_E_** | 0.91 | 0.69 | 0.31 | 0.89 | 0.76 | 0.84 | 0.73 | 0.87 | 0.95 | 0.89 | 0.82 | 0.88 | 0.84 |  |
|  | ***F*_IS_** | 0.01 | **0.25** | -0.14 | -0.05 | -0.05 | -0.01 | **0.14** | 0.02 | 0.04 | 0.02 | 0.06 | 0.00 | 0.00 | 0.03 |
|  |  |  |  |  |  |  |  |  |  |  |  |  |  |  |  |
| **Table S2** | **Contd.** |  |  |  |  |  |  |  |  |  |  |  |  |  |  |
| **Name** | **Parameters** | **Sal1** | **Sal3** | **Sal4** | **Smen05** | **Smen10** | **Spi10** | **Seb09** | **Seb25** | **Seb33** | **Seb45** | **Spi4** | **Spi6** | **Seb31** | **Overall** |
|  | ***N*_A_** | 11 | 4 | 4 | 11 | 8 | 11 | 8 | 12 | 18 | 16 | 9 | 10 | 13 |  |
|  | ***A*_R_** | 11.00 | 3.73 | 3.90 | 10.53 | 7.54 | 9.98 | 7.25 | 10.77 | 16.28 | 14.21 | 8.48 | 9.17 | 11.55 |  |
| MU11 | ***H*_O_** | 0.89 | 0.77 | 0.27 | 0.73 | 0.71 | 0.85 | 0.65 | 0.92 | 0.85 | 0.88 | 0.84 | 0.81 | 0.81 |  |
|  | ***H*_E_** | 0.90 | 0.62 | 0.28 | 0.85 | 0.72 | 0.84 | 0.71 | 0.85 | 0.94 | 0.89 | 0.83 | 0.87 | 0.86 |  |
|  | ***F*_IS_** | 0.00 | -0.24 | 0.04 | 0.14 | 0.01 | -0.01 | 0.08 | -0.09 | 0.10 | 0.01 | -0.02 | 0.07 | 0.06 | 0.01 |
|  |  |  |  |  |  |  |  |  |  |  |  |  |  |  |  |
|  | ***N*_A_** | 14 | 7 | 4 | 11 | 9 | 10 | 12 | 14 | 28 | 22 | 9 | 14 | 16 |  |
|  | ***A*_R_** | 11.63 | 5.03 | 3.15 | 9.57 | 7.27 | 8.58 | 9.61 | 10.85 | 18.74 | 16.60 | 8.13 | 11.53 | 13.41 |  |
| M1C12Q2 | ***H*_O_** | 0.92 | 0.76 | 0.24 | 0.78 | 0.69 | 0.67 | 0.78 | 0.90 | 0.83 | 0.92 | 0.73 | 0.73 | 0.73 |  |
|  | ***H*_E_** | 0.89 | 0.64 | 0.24 | 0.88 | 0.78 | 0.82 | 0.84 | 0.85 | 0.95 | 0.93 | 0.82 | 0.91 | 0.87 |  |
|  | ***F*_IS_** | -0.03 | -0.19 | -0.02 | **0.12** | 0.11 | **0.18** | 0.07 | -0.05 | **0.12** | 0.02 | 0.11 | **0.19** | **0.16** | **0.07*** |
|  |  |  |  |  |  |  |  |  |  |  |  |  |  |  |  |
|  | ***N*_A_** | 14 | 10 | 4 | 13 | 11 | 16 | 12 | 15 | 28 | 22 | 11 | 14 | 16 |  |
|  | ***A*_R_** | 11.07 | 6.83 | 3.52 | 10.38 | 7.34 | 9.57 | 8.70 | 10.83 | 17.39 | 14.69 | 7.58 | 10.16 | 11.79 |  |
| M2C12Q2 | ***H*_O_** | 0.84 | 0.71 | 0.23 | 0.82 | 0.59 | 0.81 | 0.66 | 0.84 | 0.92 | 0.88 | 0.71 | 0.70 | 0.82 |  |
|  | ***H*_E_** | 0.89 | 0.75 | 0.25 | 0.87 | 0.66 | 0.84 | 0.77 | 0.85 | 0.94 | 0.91 | 0.78 | 0.89 | 0.79 |  |
|  | ***F*_IS_** | 0.06 | 0.06 | 0.10 | 0.06 | 0.10 | 0.04 | **0.15** | 0.01 | 0.03 | 0.04 | 0.08 | **0.21** | -0.03 | **0.07*** |
|  |  |  |  |  |  |  |  |  |  |  |  |  |  |  |  |
|  | ***N*_A_** | 15 | 7 | 6 | 13 | 9 | 22 | 13 | 18 | 31 | 24 | 11 | 11 | 18 |  |
|  | ***A*_R_** | 10.76 | 4.74 | 3.43 | 10.22 | 6.86 | 10.91 | 9.30 | 11.38 | 19.23 | 13.87 | 9.01 | 8.00 | 10.57 |  |
| M3C12Q2 | ***H*_O_** | 0.87 | 0.64 | 0.25 | 0.79 | 0.71 | 0.73 | 0.82 | 0.89 | 0.89 | 0.79 | 0.79 | 0.77 | 0.68 |  |
|  | ***H*_E_** | 0.88 | 0.67 | 0.25 | 0.85 | 0.77 | 0.77 | 0.78 | 0.88 | 0.95 | 0.88 | 0.85 | 0.84 | 0.71 |  |
|  | ***F*_IS_** | 0.01 | 0.05 | 0.02 | **0.08** | 0.08 | 0.06 | -0.05 | -0.01 | **0.06** | **0.11** | **0.08** | **0.09** | 0.04 | **0.049*** |
|  |  |  |  |  |  |  |  |  |  |  |  |  |  |  |  |
|  | ***N*_A_** | 14 | 7 | 4 | 12 | 8 | 10 | 11 | 15 | 28 | 22 | 11 | 12 | 20 |  |
|  | ***A*_R_** | 11.89 | 5.77 | 3.80 | 9.71 | 6.62 | 8.80 | 9.10 | 10.19 | 19.44 | 16.55 | 8.98 | 10.49 | 15.00 |  |
| MC12Q3 | ***H*_O_** | 0.84 | 0.64 | 0.36 | 0.82 | 0.62 | 0.80 | 0.80 | 0.75 | 0.93 | 0.80 | 0.76 | 0.70 | 0.84 |  |
| **Table S2** | **Contd.** |  |  |  |  |  |  |  |  |  |  |  |  |  |  |
| **Name** | **Parameters** | **Sal1** | **Sal3** | **Sal4** | **Smen05** | **Smen10** | **Spi10** | **Seb09** | **Seb25** | **Seb33** | **Seb45** | **Spi4** | **Spi6** | **Seb31** | **Overall** |
| MC12Q3 | ***H*_E_** | 0.90 | 0.73 | 0.42 | 0.87 | 0.75 | 0.86 | 0.85 | 0.80 | 0.95 | 0.93 | 0.84 | 0.89 | 0.92 |  |
|  | ***F*_IS_** | 0.06 | 0.12 | 0.16 | 0.06 | **0.17** | 0.07 | 0.06 | 0.06 | 0.02 | **0.14** | 0.11 | **0.21** | 0.08 | **0.098*** |
|  |  |  |  |  |  |  |  |  |  |  |  |  |  |  |  |
|  | ***N*_A_** | 16 | 7 | 8 | 12 | 9 | 34 | 16 | 18 | 36 | 22 | 13 | 17 | 18 |  |
|  | ***A*_R_** | 12.24 | 5.79 | 4.18 | 10.07 | 7.04 | 17.42 | 9.56 | 12.29 | 21.11 | 15.22 | 9.71 | 11.34 | 11.88 |  |
| MSeb12WGL | ***H*_O_** | 0.90 | 0.71 | 0.27 | 0.76 | 0.70 | 0.76 | 0.81 | 0.88 | 0.97 | 0.88 | 0.84 | 0.81 | 0.89 |  |
|  | ***H*_E_** | 0.90 | 0.64 | 0.28 | 0.87 | 0.82 | 0.88 | 0.78 | 0.88 | 0.96 | 0.92 | 0.86 | 0.89 | 0.82 |  |
|  | ***F*_IS_** | 0.00 | -0.11 | 0.01 | **0.13** | **0.14** | **0.14** | -0.05 | 0.00 | -0.01 | 0.04 | 0.03 | **0.09** | -0.08 | **0.030*** |
|  |  |  |  |  |  |  |  |  |  |  |  |  |  |  |  |
|  | ***N*_A_** | 19 | 10 | 7 | 13 | 8 | 19 | 17 | 19 | 46 | 32 | 13 | 13 | 18 |  |
|  | ***A*_R_** | 14.45 | 7.08 | 4.92 | 9.01 | 6.24 | 11.10 | 11.87 | 10.81 | 24.32 | 17.95 | 9.47 | 9.63 | 12.62 |  |
| MR12Q2_6 | ***H*_O_** | 0.90 | 0.62 | 0.35 | 0.78 | 0.67 | 0.62 | 0.81 | 0.71 | 0.96 | 0.88 | 0.82 | 0.81 | 0.82 |  |
|  | ***H*_E_** | 0.93 | 0.78 | 0.38 | 0.85 | 0.79 | 0.88 | 0.89 | 0.79 | 0.97 | 0.94 | 0.83 | 0.85 | 0.88 |  |
|  | ***F*_IS_** | 0.03 | **0.20** | 0.06 | 0.08 | **0.16** | **0.29** | **0.09** | **0.11** | 0.02 | **0.07** | 0.01 | 0.06 | **0.07** | **0.095*** |
|  |  |  |  |  |  |  |  |  |  |  |  |  |  |  |  |
|  | ***N*_A_** | 17 | 5 | 4 | 15 | 10 | 41 | 13 | 20 | 35 | 20 | 15 | 14 | 19 |  |
|  | ***A*_R_** | 12.78 | 4.33 | 2.99 | 10.50 | 7.72 | 21.15 | 8.76 | 12.72 | 20.30 | 13.80 | 9.97 | 11.78 | 13.36 |  |
| MSeb12Q2 | ***H*_O_** | 0.86 | 0.55 | 0.13 | 0.86 | 0.84 | 0.98 | 0.73 | 0.89 | 0.93 | 0.97 | 0.88 | 0.72 | 0.93 |  |
|  | ***H*_E_** | 0.91 | 0.61 | 0.16 | 0.85 | 0.85 | 0.96 | 0.77 | 0.86 | 0.96 | 0.91 | 0.85 | 0.89 | 0.90 |  |
|  | ***F*_IS_** | 0.06 | 0.09 | **0.21** | -0.01 | 0.01 | -0.02 | 0.06 | -0.03 | 0.02 | -0.06 | -0.04 | **0.19** | -0.04 | 0.02 |
|  |  |  |  |  |  |  |  |  |  |  |  |  |  |  |  |
|  | ***N*_A_** | 12 | 4 | 4 | 10 | 10 | 9 | 6 | 12 | 21 | 12 | 10 | 10 | 13 |  |
|  | ***A*_R_** | 11.13 | 4.00 | 3.62 | 9.00 | 8.51 | 8.16 | 5.91 | 10.94 | 18.28 | 10.51 | 9.19 | 9.32 | 11.51 |  |
| MR12Q6 | ***H*_O_** | 0.85 | 0.68 | 0.37 | 0.78 | 0.54 | 0.71 | 0.75 | 0.78 | 0.93 | 0.79 | 0.89 | 0.74 | 0.82 |  |
|  | ***H*_E_** | 0.88 | 0.70 | 0.37 | 0.83 | 0.68 | 0.80 | 0.72 | 0.86 | 0.95 | 0.87 | 0.83 | 0.88 | 0.82 |  |
|  | ***F*_IS_** | 0.03 | 0.03 | 0.01 | 0.07 | **0.22** | 0.11 | -0.04 | 0.10 | 0.03 | 0.10 | -0.07 | **0.16** | 0.00 | **0.058*** |
|  |  |  |  |  |  |  |  |  |  |  |  |  |  |  |  |
| **Table S2** | **Contd.** |  |  |  |  |  |  |  |  |  |  |  |  |  |  |
| **Name** | **Parameters** | **Sal1** | **Sal3** | **Sal4** | **Smen05** | **Smen10** | **Spi10** | **Seb09** | **Seb25** | **Seb33** | **Seb45** | **Spi4** | **Spi6** | **Seb31** | **Overall** |
|  | ***N*_A_** | 18 | 6 | 8 | 13 | 10 | 40 | 15 | 21 | 32 | 24 | 15 | 16 | 19 |  |
|  | ***A*_R_** | 13.40 | 5.03 | 3.79 | 9.88 | 7.96 | 21.46 | 9.25 | 13.25 | 20.25 | 14.97 | 9.75 | 11.49 | 12.83 |  |
| MR12WGL | ***H*_O_** | 0.92 | 0.60 | 0.22 | 0.75 | 0.83 | 0.92 | 0.78 | 0.89 | 0.93 | 0.88 | 0.72 | 0.77 | 0.82 |  |
|  | ***H*_E_** | 0.91 | 0.64 | 0.24 | 0.86 | 0.84 | 0.95 | 0.77 | 0.87 | 0.96 | 0.92 | 0.82 | 0.88 | 0.89 |  |
|  | ***F*_IS_** | -0.01 | 0.05 | 0.05 | **0.13** | 0.01 | 0.03 | -0.01 | -0.02 | 0.03 | 0.05 | **0.12** | **0.12** | **0.07** | **0.049*** |
|  |  |  |  |  |  |  |  |  |  |  |  |  |  |  |  |
|  | ***N*_A_** | 17 | 7 | 3 | 12 | 10 | 38 | 12 | 22 | 40 | 23 | 14 | 16 | 19 |  |
|  | ***A*_R_** | 12.66 | 4.62 | 2.63 | 9.54 | 7.78 | 18.92 | 8.10 | 13.03 | 21.78 | 14.95 | 9.59 | 11.52 | 13.00 |  |
| MR12Q3 | ***H*_O_** | 0.95 | 0.62 | 0.15 | 0.75 | 0.89 | 0.93 | 0.72 | 0.77 | 0.95 | 0.93 | 0.85 | 0.78 | 0.91 |  |
|  | ***H*_E_** | 0.91 | 0.61 | 0.16 | 0.86 | 0.83 | 0.94 | 0.77 | 0.86 | 0.96 | 0.91 | 0.86 | 0.87 | 0.88 |  |
|  | ***F*_IS_** | -0.05 | -0.01 | 0.01 | **0.13** | -0.07 | 0.01 | 0.07 | **0.10** | 0.01 | -0.03 | 0.00 | **0.11** | -0.03 | 0.021 |
|  |  |  |  |  |  |  |  |  |  |  |  |  |  |  |  |
|  | ***N*_A_** | 16 | 5 | 5 | 12 | 9 | 17 | 10 | 17 | 33 | 27 | 12 | 18 | 20 |  |
|  | ***A*_R_** | 12.50 | 4.31 | 3.66 | 10.08 | 7.19 | 9.62 | 7.42 | 10.62 | 18.68 | 15.54 | 8.36 | 11.21 | 11.05 |  |
| MR12Q4 | ***H*_O_** | 0.87 | 0.56 | 0.31 | 0.82 | 0.66 | 0.74 | 0.68 | 0.77 | 0.94 | 0.89 | 0.73 | 0.73 | 0.78 |  |
|  | ***H*_E_** | 0.90 | 0.67 | 0.33 | 0.86 | 0.71 | 0.77 | 0.70 | 0.84 | 0.94 | 0.91 | 0.80 | 0.89 | 0.81 |  |
|  | ***F*_IS_** | 0.03 | **0.16** | 0.06 | 0.04 | 0.07 | 0.05 | 0.04 | 0.08 | 0.01 | 0.02 | **0.09** | **0.18** | 0.03 | **0.065*** |
|  |  |  |  |  |  |  |  |  |  |  |  |  |  |  |  |
|  | ***N*_A_** | 16 | 6 | 5 | 11 | 11 | 19 | 10 | 16 | 32 | 25 | 26 | 16 | 16 |  |
|  | ***A*_R_** | 10.83 | 4.79 | 3.53 | 9.36 | 7.75 | 10.14 | 7.49 | 10.82 | 19.34 | 14.69 | 16.60 | 10.76 | 10.94 |  |
| MR12Q5 | ***H*_O_** | 0.80 | 0.66 | 0.21 | 0.76 | 0.78 | 0.69 | 0.70 | 0.84 | 0.96 | 0.90 | 0.83 | 0.87 | 0.77 |  |
|  | ***H*_E_** | 0.88 | 0.66 | 0.23 | 0.84 | 0.75 | 0.72 | 0.72 | 0.86 | 0.95 | 0.90 | 0.94 | 0.88 | 0.84 |  |
|  | ***F*_IS_** | 0.08 | -0.01 | 0.09 | 0.09 | -0.04 | 0.05 | 0.03 | 0.03 | -0.01 | 0.00 | **0.12** | 0.00 | **0.09** | **0.038** |
|  |  |  |  |  |  |  |  |  |  |  |  |  |  |  |  |
|  | ***N*_A_** | 18 | 9 | 5 | 14 | 10 | 34 | 11 | 21 | 37 | 24 | 23 | 17 | 20 |  |
|  | ***A*_R_** | 12.26 | 6.29 | 3.53 | 10.68 | 7.65 | 15.14 | 8.07 | 12.56 | 21.43 | 13.77 | 12.91 | 9.91 | 12.66 |  |
| M-FC | ***H*_O_** | 0.89 | 0.69 | 0.30 | 0.86 | 0.79 | 0.87 | 0.66 | 0.91 | 0.92 | 0.92 | 0.88 | 0.75 | 0.77 |  |
| **Table S2** | **Contd.** |  |  |  |  |  |  |  |  |  |  |  |  |  |  |
| **Name** | **Parameters** | **Sal1** | **Sal3** | **Sal4** | **Smen05** | **Smen10** | **Spi10** | **Seb09** | **Seb25** | **Seb33** | **Seb45** | **Spi4** | **Spi6** | **Seb31** | **Overall** |
| M-FC | ***H*_E_** | 0.90 | 0.66 | 0.34 | 0.87 | 0.81 | 0.87 | 0.77 | 0.89 | 0.96 | 0.89 | 0.90 | 0.86 | 0.86 |  |
|  | ***F*_IS_** | 0.00 | -0.05 | 0.12 | 0.01 | 0.03 | -0.01 | **0.15** | -0.03 | 0.04 | -0.04 | 0.02 | **0.13** | **0.10** | 0.032 |
|  |  |  |  |  |  |  |  |  |  |  |  |  |  |  |  |
|  | ***N*_A_** | 16 | 6 | 8 | 9 | 8 | 19 | 14 | 21 | 30 | 20 |  |  |  |  |
|  | ***A*_R_** | 8.84 | 4.98 | 2.89 | 7.88 | 6.08 | 9.39 | 6.20 | 8.80 | 12.93 | 9.14 |  |  |  |  |
| Nor-Nor | ***H*_O_** | 0.78 | 0.54 | 0.44 | 0.71 | 0.63 | 0.78 | 0.78 | 0.80 | 0.78 | 0.68 |  |  |  |  |
|  | ***H*_E_** | 0.90 | 0.56 | 0.44 | 0.82 | 0.75 | 0.86 | 0.84 | 0.91 | 0.96 | 0.89 |  |  |  |  |
|  | ***F*_IS_** | **0.14** | 0.04 | 0.00 | 0.14 | 0.15 | 0.09 | **0.07** | 0.12 | **0.19** | **0.24** |  |  |  | **0.13*** |
|  |  |  |  |  |  |  |  |  |  |  |  |  |  |  |  |
|  | ***N*_A_** | 16 | 10 | 9 | 8 | 9 | 21 | 15 | 19 | 41 | 29 |  |  |  |  |
|  | ***A*_R_** | 9.45 | 3.99 | 2.40 | 7.01 | 6.82 | 12.12 | 6.54 | 8.70 | 12.72 | 9.58 |  |  |  |  |
| Nor-GL A | ***H*_O_** | 0.83 | 0.67 | 0.36 | 0.64 | 0.78 | 0.75 | 0.70 | 0.66 | 0.97 | 0.83 |  |  |  |  |
|  | ***H*_E_** | 0.91 | 0.77 | 0.40 | 0.81 | 0.82 | 0.89 | 0.84 | 0.77 | 0.96 | 0.94 |  |  |  |  |
|  | ***F*_IS_** | **0.08** | **0.13** | 0.09 | **0.21** | 0.05 | **0.16** | **0.16** | **0.15** | -0.01 | **0.11** |  |  |  | **0.11*** |
|  |  |  |  |  |  |  |  |  |  |  |  |  |  |  |  |
|  | ***N*_A_** | 17 | 9 | 9 | 9 | 10 | 20 | 15 | 19 | 38 | 26 |  |  |  |  |
|  | ***A*_R_** | 9.66 | 3.97 | 1.99 | 8.01 | 5.69 | 11.74 | 5.22 | 8.00 | 12.21 | 10.24 |  |  |  |  |
| Nor-GL B | ***H*_O_** | 0.77 | 0.60 | 0.33 | 0.79 | 0.71 | 0.76 | 0.84 | 0.80 | 0.94 | 0.83 |  |  |  |  |
|  | ***H*_E_** | 0.89 | 0.76 | 0.41 | 0.85 | 0.82 | 0.88 | 0.86 | 0.88 | 0.97 | 0.89 |  |  |  |  |
|  | ***F*_IS_** | **0.14** | **0.21** | **0.19** | **0.08** | 0.13 | **0.14** | 0.02 | 0.09 | 0.03 | **0.07** |  |  |  | **0.10*** |
|  |  |  |  |  |  |  |  |  |  |  |  |  |  |  |  |
|  | ***N*_A_** | 15 | 8 | 9 | 9 | 8 | 15 | 14 | 15 | 33 | 19 |  |  |  |  |
|  | ***A*_R_** | 8.36 | 5.52 | 4.96 | 7.60 | 4.69 | 7.83 | 3.68 | 3.12 | 11.23 | 6.97 |  |  |  |  |
| Nor-WGL | ***H*_O_** | 0.59 | 0.45 | 0.37 | 0.82 | 0.80 | 0.80 | 0.86 | 0.69 | 0.98 | 0.76 |  |  |  |  |
|  | ***H*_E_** | 0.75 | 0.67 | 0.39 | 0.85 | 0.81 | 0.89 | 0.86 | 0.83 | 0.94 | 0.84 |  |  |  |  |
|  | ***F*_IS_** | **0.21** | **0.34** | 0.07 | 0.04 | 0.01 | **0.11** | 0.00 | **0.17** | -0.04 | **0.10** |  |  |  | **0.10*** |
|  |  |  |  |  |  |  |  |  |  |  |  |  |  |  |  |
| **Table S2** | **Contd.** |  |  |  |  |  |  |  |  |  |  |  |  |  |  |
| **Name** | **Parameters** | **Sal1** | **Sal3** | **Sal4** | **Smen05** | **Smen10** | **Spi10** | **Seb09** | **Seb25** | **Seb33** | **Seb45** | **Spi4** | **Spi6** | **Seb31** | **Overall** |
|  | ***N*_A_** | 5 | 4 | 5 | 6 | 5 | 9 | 10 | 9 | 8 | 8 |  |  |  |  |
|  | ***A*_R_** | 8.62 | 4.53 | 2.30 | 7.34 | 5.92 | 6.85 | 6.11 | 6.45 | 12.19 | 8.98 |  |  |  |  |
| Nor-Giant | ***H*_O_** | 0.53 | 0.41 | 0.35 | 0.59 | 0.71 | 0.71 | 0.82 | 0.59 | 0.53 | 0.65 |  |  |  |  |
|  | ***H*_E_** | 0.69 | 0.44 | 0.37 | 0.68 | 0.79 | 0.83 | 0.91 | 0.70 | 0.83 | 0.84 |  |  |  |  |
|  | ***F*_IS_** | **0.24** | 0.06 | 0.04 | 0.14 | 0.11 | 0.16 | 0.10 | 0.16 | **0.37** | **0.24** |  |  |  | 0.17 |
|  |  |  |  |  |  |  |  |  |  |  |  |  |  |  |  |
|  | ***N*_A_** | 15 | 8 | 9 | 12 | 8 | 13 | 6 | 7 | 25 | 23 |  |  |  |  |
|  | ***A*_R_** | 8.99 | 3.16 | 4.76 | 9.84 | 3.74 | 9.01 | 4.79 | 9.27 | 9.71 | 5.49 |  |  |  |  |
| VV-Ice | ***H*_O_** | 0.83 | 0.68 | 0.77 | 0.79 | 0.66 | 0.96 | 0.51 | 0.66 | 0.92 | 0.70 |  |  |  |  |
|  | ***H*_E_** | 0.90 | 0.72 | 0.75 | 0.89 | 0.74 | 0.88 | 0.58 | 0.62 | 0.95 | 0.77 |  |  |  |  |
|  | ***F*_IS_** | 0.08 | 0.05 | -0.03 | **0.12** | 0.11 | -0.10 | 0.12 | -0.06 | 0.03 | 0.10 |  |  |  | 0.04 |
|  |  |  |  |  |  |  |  |  |  |  |  |  |  |  |  |
|  | ***N*_A_** | 11 | 8 | 6 | 10 | 7 | 10 | 5 | 4 | 19 | 14 |  |  |  |  |
|  | ***A*_R_** | 6.97 | 5.07 | 3.99 | 6.94 | 5.28 | 9.21 | 7.62 | 7.26 | 11.79 | 8.27 |  |  |  |  |
| VV-Nor | ***H*_O_** | 0.77 | 0.88 | 0.88 | 0.88 | 0.54 | 0.81 | 0.62 | 0.50 | 0.92 | 0.69 |  |  |  |  |
|  | ***H*_E_** | 0.90 | 0.73 | 0.75 | 0.89 | 0.53 | 0.85 | 0.63 | 0.46 | 0.94 | 0.73 |  |  |  |  |
|  | ***F*_IS_** | 0.15 | -0.21 | -0.18 | 0.00 | -0.01 | 0.05 | 0.02 | -0.09 | 0.02 | 0.05 |  |  |  | -0.01 |
|  |  |  |  |  |  |  |  |  |  |  |  |  |  |  |  |
|  | ***N*_A_** | 16 | 5 | 7 | 18 | 7 | 18 | 9 | 16 | 20 | 10 |  |  |  |  |
|  | ***A*_R_** | 9.02 | 4.52 | 2.74 | 7.24 | 5.35 | 5.86 | 5.89 | 6.75 | 12.63 | 8.85 |  |  |  |  |
| Fasc | ***H*_O_** | 0.82 | 0.47 | 0.53 | 0.89 | 0.44 | 0.71 | 0.67 | 0.91 | 0.84 | 0.64 |  |  |  |  |
|  | ***H*_E_** | 0.90 | 0.53 | 0.65 | 0.92 | 0.48 | 0.89 | 0.67 | 0.90 | 0.91 | 0.67 |  |  |  |  |
|  | ***F*_IS_** | 0.09 | 0.12 | 0.19 | 0.04 | 0.08 | **0.20** | 0.01 | -0.01 | **0.07** | 0.03 |  |  |  | **0.08*** |

**Table S3** Cluster names and basic data for the selected ten microsatellite loci. *N* = sample size per locus, *N*_A_ = number of alleles, *A*_R_ = corrected allelic richness, *H*_O_ and *H*_E_ = observed and expected heterozygosity, and *F*_IS_ = inbreeding coefficient . Bold *F*_IS_ values are significant at *P* = 0.05. Asterisks indicate significance after FDR control. FDR is only applied to overall *F*_IS_ values.

| **Name** | ***N*** | **Parameters** | **Sal1** | **Sal3** | **Sal4** | **Smen05** | **Smen10** | **Spi10** | **Seb09** | **Seb25** | **Seb33** | **Seb45** | **Overall** |
| --- | --- | --- | --- | --- | --- | --- | --- | --- | --- | --- | --- | --- | --- |
|  |  | ***N*_A_** | 16 | 5 | 7 | 16 | 6 | 9 | 9 | 16 | 19 | 9 |  |
|  |  | ***A*_R_** | 9.05 | 3.05 | 4.74 | 9.58 | 3.46 | 6.19 | 4.61 | 9.23 | 9.71 | 5.20 | 6.48 |
| Fasciatus | 44 | ***H*_O_** | 0.82 | 0.48 | 0.55 | 0.89 | 0.43 | 0.75 | 0.66 | 0.91 | 0.90 | 0.64 |  |
|  |  | ***H*_E_** | 0.90 | 0.51 | 0.64 | 0.92 | 0.46 | 0.79 | 0.66 | 0.90 | 0.90 | 0.65 |  |
|  |  | ***F*_IS_** | 0.10 | 0.06 | 0.15 | 0.03 | 0.05 | 0.05 | 0.00 | -0.01 | -0.01 | 0.02 | **0.04** |
|  |  |  |  |  |  |  |  |  |  |  |  |  |  |
|  |  | ***N*_A_** | 5 | 3 | 7 | 7 | 6 | 6 | 11 | 10 | 19 | 8 |  |
|  |  | ***A*_R_** | 3.21 | 2.15 | 3.69 | 5.55 | 4.32 | 3.66 | 7.06 | 5.94 | 9.15 | 5.24 | 5.00 |
| Giants | 51 | ***H*_O_** | 0.46 | 0.34 | 0.35 | 0.73 | 0.76 | 0.67 | 0.90 | 0.72 | 0.89 | 0.68 |  |
|  |  | ***H*_E_** | 0.51 | 0.34 | 0.40 | 0.75 | 0.76 | 0.61 | 0.85 | 0.72 | 0.89 | 0.70 |  |
|  |  | ***F*_IS_** | **0.11** | 0.00 | 0.13 | 0.03 | 0.01 | -0.09 | -0.05 | -0.01 | 0.00 | 0.02 | 0.01 |
|  |  |  |  |  |  |  |  |  |  |  |  |  |  |
|  |  | ***N*_A_** | 13 | 7 | 3 | 6 | 7 | 6 | 9 | 7 | 26 | 20 |  |
|  |  | ***A*_R_** | 8.51 | 3.95 | 2.14 | 5.01 | 4.82 | 4.39 | 4.41 | 4.14 | 11.66 | 9.10 | 5.81 |
| Norvegicus-A | 50 | ***H*_O_** | 0.91 | 0.76 | 0.24 | 0.80 | 0.69 | 0.60 | 0.78 | 0.52 | 0.98 | 0.82 |  |
|  |  | ***H*_E_** | 0.89 | 0.67 | 0.30 | 0.76 | 0.73 | 0.71 | 0.69 | 0.55 | 0.95 | 0.90 |  |
|  |  | ***F*_IS_** | -0.02 | -0.14 | 0.21 | **-0.05** | 0.05 | 0.15 | -0.13 | 0.05 | -0.03 | 0.09 | 0.01 |
|  |  |  |  |  |  |  |  |  |  |  |  |  |  |
|  |  | ***N*_A_** | 13 | 5 | 7 | 7 | 8 | 10 | 10 | 17 | 29 | 12 |  |
|  |  | ***A*_R_** | 8.21 | 3.86 | 4.37 | 5.36 | 5.75 | 6.80 | 6.60 | 9.89 | 12.85 | 6.62 | 7.03 |
| Norvegicus-B | 50 | ***H*_O_** | 0.91 | 0.57 | 0.48 | 0.72 | 0.74 | 0.82 | 0.74 | 0.88 | 0.96 | 0.80 |  |
|  |  | ***H*_E_** | 0.87 | 0.65 | 0.51 | 0.79 | 0.80 | 0.84 | 0.82 | 0.92 | 0.96 | 0.82 |  |
|  |  | ***F*_IS_** | -0.04 | 0.12 | 0.05 | 0.09 | 0.08 | 0.02 | 0.09 | 0.05 | 0.00 | 0.02 | 0.05 |
|  |  |  |  |  |  |  |  |  |  |  |  |  |  |
| Table S3 | Contd. |  |  |  |  |  |  |  |  |  |  |  |  |
| **Name** | ***N*** | **Parameters** | **Sal1** | **Sal3** | **Sal4** | **Smen05** | **Smen10** | **Spi10** | **Seb09** | **Seb25** | **Seb33** | **Seb45** | **Overall** |
|  |  | ***N*_A_** | 17 | 5 | 3 | 8 | 9 | 16 | 11 | 18 | 32 | 15 |  |
|  |  | ***A*_R_** | 10.05 | 3.93 | 2.20 | 6.61 | 6.23 | 8.72 | 6.74 | 8.60 | 12.85 | 9.12 | 7.50 |
| ‘deep’ Mentella | 41 | ***H*_O_** | 0.95 | 0.53 | 0.20 | 0.69 | 0.90 | 0.83 | 0.83 | 0.80 | 0.95 | 0.90 |  |
|  |  | ***H*_E_** | 0.92 | 0.57 | 0.18 | 0.84 | 0.83 | 0.88 | 0.84 | 0.84 | 0.96 | 0.90 |  |
|  |  | ***F*_IS_** | -0.03 | 0.08 | -0.07 | **0.18** | -0.08 | 0.06 | 0.02 | 0.04 | 0.01 | 0.00 | 0.03 |
|  |  |  |  |  |  |  |  |  |  |  |  |  |  |
|  |  | ***N*_A_** | 14 | 6 | 4 | 13 | 8 | 13 | 6 | 14 | 24 | 21 |  |
|  |  | ***A*_R_** | 8.03 | 4.16 | 2.52 | 7.48 | 5.23 | 7.58 | 4.37 | 8.39 | 11.57 | 9.97 | 6.93 |
| ‘slope’ Mentella | 45 | ***H*_O_** | 0.80 | 0.76 | 0.20 | 0.91 | 0.62 | 0.78 | 0.71 | 0.76 | 0.93 | 0.89 |  |
|  |  | ***H*_E_** | 0.87 | 0.70 | 0.26 | 0.86 | 0.66 | 0.86 | 0.66 | 0.86 | 0.95 | 0.91 |  |
|  |  | ***F*_IS_** | 0.09 | -0.08 | 0.23 | -0.07 | 0.05 | 0.10 | -0.08 | 0.12 | 0.01 | 0.02 | 0.03 |
|  |  |  |  |  |  |  |  |  |  |  |  |  |  |
|  |  | ***N*_A_** | 18 | 7 | 4 | 13 | 8 | 12 | 10 | 12 | 29 | 18 |  |
|  |  | ***A*_R_** | 9.18 | 5.33 | 2.89 | 7.21 | 5.52 | 7.08 | 5.74 | 6.55 | 12.65 | 7.92 | 7.01 |
| ‘shallow’ Mentella | 45 | ***H*_O_** | 0.89 | 0.80 | 0.36 | 0.80 | 0.80 | 0.82 | 0.69 | 0.87 | 0.98 | 0.87 |  |
|  |  | ***H*_E_** | 0.90 | 0.74 | 0.36 | 0.84 | 0.79 | 0.84 | 0.64 | 0.83 | 0.96 | 0.85 |  |
|  |  | ***F*_IS_** | 0.02 | -0.08 | 0.01 | 0.05 | -0.01 | 0.03 | -0.07 | -0.05 | -0.02 | -0.02 | -0.01 |
|  |  |  |  |  |  |  |  |  |  |  |  |  |  |
|  |  | ***N*_A_** | 12 | 8 | 7 | 12 | 7 | 19 | 6 | 8 | 23 | 17 |  |
|  |  | ***A*_R_** | 8.50 | 5.24 | 5.30 | 8.01 | 4.52 | 10.38 | 3.68 | 3.71 | 11.52 | 6.75 | 6.76 |
| Viviparus | 44 | ***H*_O_** | 0.88 | 0.75 | 0.84 | 0.86 | 0.50 | 0.86 | 0.50 | 0.59 | 0.95 | 0.68 |  |
|  |  | ***H*_E_** | 0.90 | 0.73 | 0.77 | 0.88 | 0.62 | 0.93 | 0.60 | 0.58 | 0.94 | 0.72 |  |
|  |  | ***F*_IS_** | 0.02 | -0.02 | -0.10 | 0.02 | 0.19 | 0.07 | 0.16 | -0.02 | -0.01 | 0.05 | 0.03 |
|  |  |  |  |  |  |  |  |  |  |  |  |  |  |
|  |  | ***N*_A_** | 8 | 4 | 4 | 8 | 3 | 7 | 4 | 5 | 10 | 5 |  |
|  |  | ***A*_R_** | 8.00 | 4.00 | 4.00 | 8.00 | 3.00 | 7.00 | 4.00 | 5.00 | 10.00 | 5.00 | 5.80 |
| ‘shallow’ X Fasciatus | 6 | ***H*_O_** | 1.00 | 0.67 | 0.33 | 1.00 | 0.67 | 0.83 | 0.67 | 1.00 | 1.00 | 0.67 |  |
| Table S3 | Contd. |  |  |  |  |  |  |  |  |  |  |  |  |
| **Name** | ***N*** | **Parameters** | **Sal1** | **Sal3** | **Sal4** | **Smen05** | **Smen10** | **Spi10** | **Seb09** | **Seb25** | **Seb33** | **Seb45** | **Overall** |
| ‘shallow’ X Fasciatus |  | ***H*_E_** | 0.92 | 0.71 | 0.70 | 0.92 | 0.53 | 0.91 | 0.76 | 0.79 | 0.97 | 0.74 |  |
|  |  | ***F*_IS_** | -0.09 | 0.07 | 0.55 | -0.09 | -0.29 | 0.09 | 0.13 | -0.30 | -0.03 | 0.11 | 0.02 |
|  |  |  |  |  |  |  |  |  |  |  |  |  |  |
|  |  | ***N*_A_** | 17 | 7 | 4 | 12 | 10 | 16 | 11 | 18 | 38 | 24 |  |
|  |  | ***A*_R_** | 9.23 | 5.14 | 2.47 | 7.99 | 6.21 | 8.29 | 7.43 | 8.15 | 13.15 | 9.79 | 7.79 |
| ‘shallow’ X Norvegicus-B | 60 | ***H*_O_** | 0.73 | 0.71 | 0.23 | 0.86 | 0.81 | 0.82 | 0.83 | 0.85 | 0.95 | 0.92 |  |
|  |  | ***H*_E_** | 0.91 | 0.76 | 0.23 | 0.88 | 0.82 | 0.88 | 0.84 | 0.85 | 0.96 | 0.91 |  |
|  |  | ***F*_IS_** | **0.20** | 0.06 | -0.02 | 0.02 | 0.00 | **0.07** | 0.01 | 0.00 | 0.02 | -0.01 | **0.04** |
|  |  |  |  |  |  |  |  |  |  |  |  |  |  |
|  |  | ***N*_A_** | 14 | 6 | 3 | 11 | 10 | 14 | 11 | 15 | 27 | 16 |  |
|  |  | ***A*_R_** | 9.13 | 4.24 | 2.20 | 6.89 | 6.48 | 8.58 | 6.71 | 7.74 | 12.23 | 9.58 | 7.38 |
| ‘deep’ X ‘shallow’ | 45 | ***H*_O_** | 0.91 | 0.69 | 0.20 | 0.73 | 0.84 | 0.82 | 0.82 | 0.78 | 1.00 | 0.93 |  |
|  |  | ***H*_E_** | 0.91 | 0.65 | 0.19 | 0.83 | 0.84 | 0.89 | 0.82 | 0.82 | 0.96 | 0.91 |  |
|  |  | ***F*_IS_** | 0.00 | -0.06 | -0.07 | 0.13 | 0.00 | 0.08 | -0.01 | 0.06 | -0.05 | -0.02 | 0.01 |
|  |  |  |  |  |  |  |  |  |  |  |  |  |  |
|  |  | ***N*_A_** | 12 | 6 | 3 | 12 | 9 | 10 | 6 | 7 | 22 | 14 |  |
|  |  | ***A*_R_** | 8.69 | 4.31 | 2.17 | 7.93 | 6.40 | 7.36 | 5.29 | 5.40 | 12.24 | 9.37 | 6.92 |
| ‘deep’ X ‘slope’ | 22 | ***H*_O_** | 0.82 | 0.55 | 0.09 | 0.95 | 0.77 | 0.91 | 0.68 | 0.82 | 0.91 | 0.86 |  |
|  |  | ***H*_E_** | 0.89 | 0.59 | 0.17 | 0.86 | 0.79 | 0.87 | 0.75 | 0.77 | 0.95 | 0.90 |  |
|  |  | ***F*_IS_** | 0.08 | 0.07 | 0.48 | -0.12 | 0.03 | -0.05 | 0.09 | -0.07 | 0.05 | 0.05 | **0.02** |
|  |  |  |  |  |  |  |  |  |  |  |  |  |  |
|  |  | ***N*_A_** | 13 | 6 | 3 | 10 | 7 | 12 | 4 | 10 | 23 | 14 |  |
|  |  | ***A*_R_** | 10.12 | 5.03 | 2.96 | 8.24 | 5.65 | 8.52 | 3.83 | 6.79 | 13.15 | 9.06 | 7.34 |
| ‘shallow’ X Viviparus | 18 | ***H*_O_** | 0.89 | 0.72 | 0.39 | 0.83 | 0.56 | 0.83 | 0.67 | 0.67 | 0.94 | 0.83 |  |
|  |  | ***H*_E_** | 0.93 | 0.75 | 0.48 | 0.90 | 0.79 | 0.89 | 0.65 | 0.80 | 0.96 | 0.89 |  |
|  |  | ***F*_IS_** | 0.05 | 0.03 | 0.20 | **0.07** | **0.31** | 0.06 | -0.03 | 0.18 | 0.02 | 0.06 | **0.09*** |
|  |  |  |  |  |  |  |  |  |  |  |  |  |  |
| Table S3 | Contd. |  |  |  |  |  |  |  |  |  |  |  |  |
| **Name** | ***N*** | **Parameters** | **Sal1** | **Sal3** | **Sal4** | **Smen05** | **Smen10** | **Spi10** | **Seb09** | **Seb25** | **Seb33** | **Seb45** | **Overall** |
|  |  | ***N*_A_** | 10 | 6 | 7 | 7 | 7 | 9 | 10 | 13 | 21 | 14 |  |
|  |  | ***A*_R_** | 8.20 | 4.77 | 4.48 | 6.19 | 6.16 | 7.28 | 7.73 | 8.01 | 13.27 | 9.60 | 7.57 |
| Norvegicus- AXB | 17 | ***H*_O_** | 0.75 | 0.82 | 0.53 | 0.75 | 0.88 | 0.82 | 0.71 | 0.76 | 1.00 | 0.94 |  |
|  |  | ***H*_E_** | 0.88 | 0.70 | 0.49 | 0.83 | 0.83 | 0.87 | 0.85 | 0.82 | 0.97 | 0.90 |  |
|  |  | ***F*_IS_** | 0.16 | -0.18 | -0.08 | 0.10 | -0.06 | 0.06 | **0.17** | 0.07 | -0.03 | -0.05 | **0.02** |

**Figures**

**Fig. S1:** Identification of number of genetic clusters among *S. mentella* samples using Evanno method (Evanno et al. 2005). A total of three genetic clusters were suggested for the data.

**Fig. S2:** Identification of number of genetic clusters among the 35 *Sebastes* samples using Evanno method (Evanno et al. 2005). A total of six genetic clusters were suggested for the data.

**Fig. S3:** Distribution of Q values (Admixture proportions: Y-axis) and their associated 90% probability intervals for individuals of the ‘shallow’ and ‘deep’ clusters from Greenland and adjacent waters. The values have been ranked starting from zero (Pure ‘deep’) to one (Pure ‘shallow’) on the X-axis; (a) the observed, (b) simulation with a mechanical mixing and (c) the hybrid swarm situations have been illustrated.

**Fig. S4:** Distribution of Q values (Admixture proportions: Y-axis) and their associated 90% probability intervals for individuals of the ‘deep’ and ‘slope’ clusters from Greenland and adjacent waters. The values have been ranked starting from zero (Pure ‘deep’) to one (Pure ‘slope’) on the X-axis; (a) the observed, (b) simulation with a mechanical mixing and (c) the hybrid swarm situations have been illustrated.

**Fig. S5:** Distribution of Q values (Admixture proportions: Y-axis) and their associated 90% probability intervals for individuals of the ‘shallow’ and ‘slope’ clusters from Greenland and adjacent waters. The values have been ranked starting from zero (Pure ‘shallow’) to one (Pure ‘slope’) on the X-axis; (a) the observed, (b) simulation with a mechanical mixing and (c) the hybrid swarm situations have been illustrated.

**Fig. S6:** Distribution of Q values (Admixture proportions: Y-axis) and their associated 90% probability intervals for individuals of the ‘Norvegicus-A’ and ‘deep’ clusters from Greenland and adjacent waters. The values have been ranked starting from zero (Pure ‘Norvegicus-A’) to one (Pure ‘deep’) on the X-axis; (a) the observed, (b) simulation with a mechanical mixing and (c) the hybrid swarm situations have been illustrated.

**Fig. S7:** Distribution of Q values (Admixture proportions: Y-axis) and their associated 90% probability intervals for individuals of the ‘Viviparus’ and ‘slope’ clusters from Greenland and adjacent waters. The values have been ranked starting from zero (Pure ‘Viviparus’) to one (Pure ‘slope’) on the X-axis; (a) the observed, (b) simulation with a mechanical mixing and (c) the hybrid swarm situations have been illustrated.

**Fig. S8:** Distribution of Q values (Admixture proportions: Y-axis) and their associated 90% probability intervals for individuals of the ‘Norvegicus-B’ and ‘shallow’ clusters from the Northeast Arctic waters. The values have been ranked starting from zero (Pure ‘Norvegicus-B’) to one (Pure ‘shallow’) on the X-axis; (a) the observed, (b) simulation with a mechanical mixing and (c) the hybrid swarm situations have been illustrated.

**Fig. S9:** Distribution of Q values (Admixture proportions: Y-axis) and their associated 90% probability intervals for individuals of the ‘shallow’ and ‘Viviparus’ clusters from the Northeast Arctic waters. The values have been ranked starting from zero (Pure ‘shallow’) to one (Pure ‘Viviparus’) on the X-axis; (a) the observed, (b) simulation with a mechanical mixing and (c) the hybrid swarm situations have been illustrated.

**Fig. S10:** Distribution of Q values (Admixture proportions: Y-axis) and their associated 90% probability intervals for individuals of the ‘Norvegicus-B’ and ‘Viviparus’ clusters from the Northeast Arctic waters. The values have been ranked starting from zero (Pure ‘Norvegicus-B’) to one (Pure ‘Viviparus’) on the X-axis; (a) the observed, and (b) simulation with a mechanical mixing situations have been illustrated. Since hybridization was clearly infrequent, the scenario was not compared with a hybrid swarm.

**Fig. S11:** Distribution of Q values (Admixture proportions: Y-axis) and their associated 90% probability intervals for individuals of the ‘shallow’ and ‘deep’ clusters from the Northwest Atlantic waters. The values have been ranked starting from zero (Pure ‘shallow’) to one (Pure ‘deep’) on the X-axis; (a) the observed, (b) simulation with a mechanical mixing and (c) the hybrid swarm situations have been illustrated.

**Fig. S12:** Distribution of Q values (Admixture proportions: Y-axis) and their associated 90% probability intervals for individuals of the ‘Fasciatus’ and ‘shallow’ clusters from the Northwest Atlantic waters. The values have been ranked starting from zero (Pure ‘Fasciatus’) to one (Pure ‘shallow’) on the X-axis; (a) the observed, and (b) simulation with a mechanical mixing situations have been illustrated. Since hybridization was clearly infrequent, the scenario was not compared with a hybrid swarm.

**Fig. S13:** Distribution of Q values (Admixture proportions: Y-axis) and their associated 90% probability intervals for individuals of the ‘deep’ and ‘Fasciatus’ clusters from the Northwest Atlantic waters. The values have been ranked starting from zero (Pure ‘deep’) to one (Pure ‘Fasciatus’) on the X-axis; (a) the observed, and (b) simulation with a mechanical mixing situations have been illustrated.

**Fig. S14:** Distribution of Q values (Admixture proportions: Y-axis) and their associated 90% probability intervals for individuals of the ‘Fasciatus’ and ‘Norvegicus-B’ clusters from the Northwest Atlantic waters. The values have been ranked starting from zero (Pure ‘Fasciatus’) to one (Pure ‘Norvegicus-B’) on the X-axis; (a) the observed, and (b) simulation with a mechanical mixing situations have been illustrated.

**Fig. S15:** Distribution of Q values (Admixture proportions: Y-axis) and their associated 90% probability intervals for individuals of the ‘Norvegicus-B’ and ‘deep’ clusters from the Northwest Atlantic waters. The values have been ranked starting from zero (Pure ‘Norvegicus-B’) to one (Pure ‘deep’) on the X-axis; (a) the observed, and (b) simulation with a mechanical mixing situations have been illustrated.

**Fig. S16:** Distribution of Q values (Admixture proportions: Y-axis) and their associated 90% probability intervals for individuals of the ‘Norvegicus-B’ and ‘shallow’ clusters from the Northwest Atlantic waters. The values have been ranked starting from zero (Pure ‘Norvegicus-B’) to one (Pure ‘shallow’) on the X-axis; (a) the observed, and (b) simulation with a mechanical mixing situations have been illustrated.

**Fig. S17:** Comparison of Q values for real and simulated (sim) baseline individuals (Test for simulation bias). The values have been ranked starting from zero (Pure ‘shallow’) to one (Pure ‘deep’) on the X-axis.
